# Advances in experimental and computational methodologies for the study of microbial-surface interactions at different omics levels

**DOI:** 10.3389/fmicb.2022.1006946

**Published:** 2022-11-28

**Authors:** Juan José González-Plaza, Cristina Furlan, Tomaž Rijavec, Aleš Lapanje, Rocío Barros, Juan Antonio Tamayo-Ramos, Maria Suarez-Diez

**Affiliations:** ^1^International Research Centre in Critical Raw Materials-ICCRAM, University of Burgos, Burgos, Spain; ^2^Laboratory of Systems and Synthetic Biology, Wageningen University and Research, Wageningen, Netherlands; ^3^Department of Environmental Sciences, Jožef Stefan Institute, Ljubljana, Slovenia

**Keywords:** biofilms, genomics, transcriptomics, metabolomics, proteomics, omics data integration, systems biology

## Abstract

The study of the biological response of microbial cells interacting with natural and synthetic interfaces has acquired a new dimension with the development and constant progress of advanced omics technologies. New methods allow the isolation and analysis of nucleic acids, proteins and metabolites from complex samples, of interest in diverse research areas, such as materials sciences, biomedical sciences, forensic sciences, biotechnology and archeology, among others. The study of the bacterial recognition and response to surface contact or the diagnosis and evolution of ancient pathogens contained in archeological tissues require, in many cases, the availability of specialized methods and tools. The current review describes advances in *in vitro* and *in silico* approaches to tackle existing challenges (e.g., low-quality sample, low amount, presence of inhibitors, chelators, etc.) in the isolation of high-quality samples and in the analysis of microbial cells at genomic, transcriptomic, proteomic and metabolomic levels, when present in complex interfaces. From the experimental point of view, tailored manual and automatized methodologies, commercial and in-house developed protocols, are described. The computational level focuses on the discussion of novel tools and approaches designed to solve associated issues, such as sample contamination, low quality reads, low coverage, etc. Finally, approaches to obtain a systems level understanding of these complex interactions by integrating multi omics datasets are presented.

## Introduction

Cell interaction with biotic and abiotic surfaces involves complex processes including locating, approaching, and sensing the proximity of the surface. On occasion, the interaction may be initiated through random deposition, as exemplified by viruses that are not actively performing any type of biological processes beyond cell infection. Yet for cells other than viruses this interaction is actively sought as a number of advantages are present when comparing surface attached growth and planktonic lifestyle, and these include among others an improved access to nutrients and better chances for survival (Tuson and Weibel, 2013). Plant surfaces interacting with bacteria are an example of a type of interaction between microbiome and a biotic material (part of a host) that provides benefits for both players (Pieterse et al., 2016). Therefore, interaction with surfaces provides evolutionary advantage as it increases the odds of survival for the microbial species in the long run.

Microorganisms are often found spatially and functionally organized in 3D aggregated structures, which can be suspended in the liquid as flocs and granule-like aggregates, or they can be attached to a surface in the form of biofilms and mats (Stolz, 2000). Biofilms are one and a recalcitrant expression of microbial adaptation and survival to desiccation, stress, or harsh environments. Biological evolutionary pressures have selected the development of this and other complex biological systems as a microenvironment where the “self” has in certain ways been lost for the community purpose of survival and optimization of resources (Hug and Co, 2018).

When considering man-made surfaces, microbial-surface interactions can start even before cell attachment. Upon exposure to the environment, the raw surface gets covered by organic materials forming a conditioning film (Berne et al., 2018). This makes the surface more familiar to microbes allowing a better interaction, adhesion, or active attachment. It has been demonstrated that not all bacteria are part of the group of primary colonizers, but can only attach once certain other bacterial species have established themselves in the community, as reviewed in Little and Wagner, (1997). The initial community starts to grow in size and complexity through time producing ever more complex surface structures, i.e., physically (different 3D shapes), physiologically (increased metabolic activity), and compositionally (higher phylogenetic diversity). The conceptual model of biofilm formation has been debated for decades (Monds and O’Toole, 2009), and only now the methodology is allowing us to tap deep into the chemical and physical interactions that cells have with different surfaces and with one another as the biofilm is maturing. Colonization is started by individual planktonic cells that land or deposit over a given surface, leading to the colonization stage in the biofilm forming process (Li et al., 2020). Biochemical pathways associated with the biofilm lifestyle are triggered including alteration of the membrane lipid profile (Favre et al., 2018; Tang et al., 2021). These modifications are probably owed to the need of a continuous flow of information between the biofilm components, and a lower requirement for defense against the outer environment than during a planktonic lifestyle. Some other specific traits in the biofilm lifestyle indicate that bacteria use siderophores (secondary metabolites) to capture preferentially available iron (Guo et al., 2021). Overall, at a mature stage, these structures present a variety of microniches and environments subject to stresses that induce the development of responsive and adaptive mechanisms (Chavez-Dozal et al., 2015).

Cellular attachment events are of particular interest as in many cases, microbial surface colonization results in unwanted outcomes (Flemming et al., 2016). For instance, biofouling negatively affects the flow of liquids in pipes (Polman et al., 2013, 2020), leading to the damage of surfaces (Coetser and Cloete, 2005). Therefore, cell-surface interactions can pose economic losses of a considerable entity when expensive antifouling treatments have to be applied (Flemming, 2020). Other issues include the colonization of biotic tissues and hospital surfaces or devices causing medical complications (Harding and Reynolds, 2014; Russotto et al., 2015; Li et al., 2020). Detrimental microorganisms can thrive in a myriad of clinical surfaces (Cobrado et al., 2017), posing a serious risk to become a reservoir of hospital acquired infections (Boyce, 2016; Gostine et al., 2020). More specifically, the colonization of medical devices that break the physical boundaries between the external environment and the internal human body space, leads to severe and life-threatening consequences for patients at Intensive Care Units (Vickery et al., 2012; Vitális et al., 2020; Zhang et al., 2021). Presence of certain strains is not only critical in the regular clinical practice, but has been appointed as a very important aspect to monitor and control in upcoming space exploration (Mahnert et al., 2021), or crewed Moon or Mars missions where dysbiosis could be enhanced by the absence of compensating microbiomes. Metabolic transformations due to the newly established community or physiological processes from single cells can generate toxic products in drinking water, food and chemical production lines (Bachmann and Edyvean, 2005; Dupre et al., 2019), but also affect the viability of cultural heritage (Cennamo et al., 2016; Marvasi et al., 2019). The year 2020 has shown how exposed and vulnerable we are as a globalized species, where infectious agents can cross the globe in a matter of hours. The spread of those agents can be facilitated by deposition at high-touch surfaces (Cassidy et al., 2020). Finally, cell-surface interactions in a mature biofilm show increased recalcitrance to cleaning or toward antimicrobial treatments. This resistance is achieved by genetic mechanisms in combination with specific structural properties of the biofilm such as diminished antibiotic penetration due to the extracellular matrix (Gilbert et al., 1997).

For all the above, an improved understanding of the succession of phenotypes taking place when cells interact with surfaces is of paramount importance. Detection of microbial population shifts, genes, transcripts, proteins, or metabolites indicating a seeding phase (Monge et al., 2021) is essential for managing strategies, to evaluate establishment risks, and to judge the possibility of success of interventions. Control of microbial interactions with surfaces is also relevant for biotechnological purposes. For instance, microorganisms can be used as a tool for the detoxification of pollutants present in industrial wastewater, for a more efficient and controllable production of biomolecules of industrial interest, or the production of added-value surfaces (e.g., a nanomaterial with a defined beneficial biological layer of microorganisms that prevents the establishment of pathogenic or unwanted strains) (Deev et al., 2021).

Molecular biology methods enable investigation on how the cell senses and responds to surfaces, both on the community and on the single-cell levels. Genomics, transcriptomics, proteomics and metabolomics, collectively called “omics” technologies (Beale et al., 2016), cover the full range of aspects and inform on changes on microbial molecular phenotypes induced by the contact with the surface (Seneviratne et al., 2020).

These methodologies, still under development, are already providing answers to specific questions related among others to: (i) mechanisms for active microbial attachment, (ii) composition of surface interacting microbial communities, (iii) specific molecular phenotypes exhibited prior and during interaction with the surface, (iv) surface modifications that decrease potential biofilm formation, and (v) surface modifications to selectively promote cellular attachment.

Notwithstanding, several main challenges need to be addressed to advance the study of cell-surface interaction. *Time distribution:* it is crucial to understand the time span and resolution with which to study such a continuous process characterized by a succession of molecular phenotypes. *Spatial distribution:* spatial gradients as a result of biofilm formation this result in differential environments with chemical features that may represent a burden in the isolation of certain materials due to impaired penetration of reagents. *Low quantity of material:* surfaces are usually covered only by a low quantity of viable cells, specially at early stages of biofilm development. *Complexity of the extracellular matr*ix: DNA, proteins, and other cellular metabolites can irreversibly bind to the extracellular matrix, which is piling up on the exposed surfaces and is entrapping the cells. This matrix often represents a problem for successful isolation of biological material. *Data analysis*: computational methods tailored to the specific data type are required.

**Figure 1:**
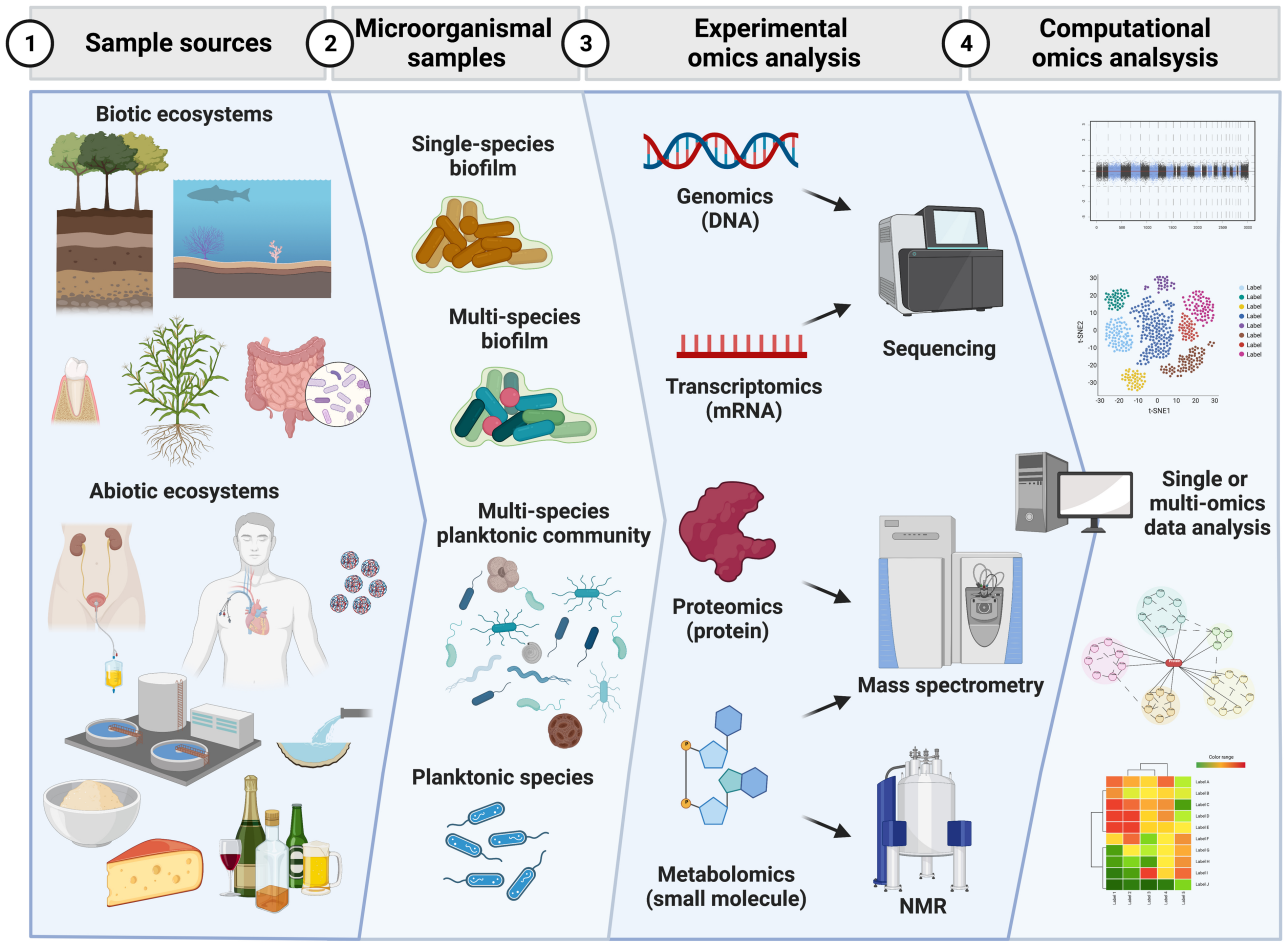
Workflow of major steps in omics analysis of microorganismal samples. Initial specimens can be obtained from biotic (e.g., plants, animals) or abiotic (e.g., medical devices, food, pipes, water) ecosystems. In these sources microorganismal samples can be present as planktonic and biofilm growing communities of a single or multiple species. From the microorganismal samples single or multiple omics experiments can be conducted by extracting singly or simultaneously genomic DNA, messenger RNA, proteins or chemical small molecules (metabolites). Genomics and transcriptomics experiments have third generation sequencers as end point analytical instruments to produce data while proteomics and metabolomics use mass spectrometry as end technology. Metabolomics also takes advantage of nuclear magnetic resonance (NMR) measurements. The huge amount of data can then be analyzed in a separate or integrative approach. Data analysis approaches based on statistics, complexity reduction, classification and prediction *via* machine learning tools are used to interpret the rich amount of data obtained and answer and generate new hypotheses. Created with BioRender.com.

Considering the presented challenges, this review highlights the current wet and dry (*in silico*) methodologies in the four main omics approaches: genomics, transcriptomics, proteomics, and metabolomics (Figure 1).

We first present a methodological perspective, attending to the intrinsic difficulties encountered at each omics level from sample recovery to the use of solvents to selectively capture molecules of interest. We also reflect on the potential of recent advances in omics data acquisition, such as long read sequencing technology, for the study of complex communities. From a data analysis perspective, we comment on pipelines to analyze and interpret the obtained data. Lastly, we discuss systems level approaches that integrate multiple datasets and data types to arrive at a holistic understanding of the system.

## Generation and analysis of genomics data for the study of microorganisms in complex matrices

Genome encoded functional analysis can accurately characterize the functions and the interaction among microbes and with their environment. Moreover, DNA sequence analysis in complex metagenomic samples informs on the precise composition of the community and the possible roles of its members. Sequencing of ubiquitous marker genes such as those associated with the small subunit of ribosomal RNA, 16S for prokaryotes, 18S for eukaryotes or internal transcribed spacers (ITS) for fungi, or the cytochrome c-oxidase (EC 1.9. 3.1), is an efficient approach for community profiling that has been applied to explore a broad range of environments, such as the human microbiome, marine communities, or other environmental samples including soil (Bharti and Grimm, 2021). Genomics is especially well suited for studies where the samples of interest are sensitive, such as ancient cultural heritage items (González and Sáiz-Jiménez, 2005; Imperi et al., 2007; Bharti and Grimm, 2021), although other minimally invasive approaches have been developed for these cases (Marvasi et al., 2019). Decorative elements developed in pre-industrial ages represent a great environment for microbial growth due to its composition that can include animal fatty acids and protein substrates (Cennamo et al., 2016).

### Sample preparation

The biomass recovery step is a major concern when working with cells growing over a surface. In biofilms, the complex extracellular matrix requires methods for detachment of cells, otherwise the extraction methods will yield less nucleic acids. A study aimed to understand the dynamics of biofilm dispersal (de Vries et al., 2021), used an approach of detachment and dissolution of the biofilm structure grown on filter membranes by strong bead-beating at 50,000 rpm during a short period of time having used sterile milli-Q water as a solvent. Authors succeeded in achieving efficient detachment of cells from their matrix. Disruption of cells is of paramount importance when working with low-biomass samples, and efficient lysis is carried out as with stronger mechanical methods by adding ceramic beads in the tubes used by the instrument (Rychert et al., 2021). Recovery of biofouling communities is also a complex procedure as shown by Kim et al. (2021). Their process includes a step to remove invertebrates and macroalgae from the exposed metal surface, microbial communities are then washed and recovered by rubber spatula scraping. A filtration step (3 μm pore) was intended for removal of organic particles, coupled with another step of filtration (0.2 μm pore) for microbial biomass collection prior to DNA isolation using standard commercial kits.

These approaches illustrate the necessary phases for DNA isolation and extraction from samples originating from complex environments or matrices, and how it is challenged by the presence of proteins, metal ions, and other impurities. In that regard, lysozyme and proteinase pre-lysis treatments have been used to target extracellular contaminants, as in the study of *L. monocytogenes* isolates obtained from meat factories surfaces (Nastasijevic et al., 2017). Moreover, samples with low microbial biomass can be even more sensitive to these impurities. Complex polysaccharides, such as those found on the extracellular matrix, are known to inhibit PCR (Schrader et al., 2012). Some approaches in biofilm research have applied steps of enzymatic digestion aimed to diminish potential matrix contaminants (Leventhal et al., 2018). Contaminants and impurities impair enzymatic amplification, which can cause major biases that are particularly relevant when analyzing metagenomics samples (Angelakis et al., 2016). Similarly, metal ions affect DNA extraction through inhibitory effects, which is especially important in archeological and/or forensic samples where calcium-induced inhibition plays a major role (Kuffel et al., 2021). Specific protocols have been developed for these samples, relying on the use of chelators for mineral removal (Balayan et al., 2015), or biofilm dedicated commercial kits for DNA isolation (Paix et al., 2020).

On some occasions, low biomass recovery is imposed by the intrinsic limitations of the studied surface, namely when approaching heritage artifacts or monuments under special protection for which a maximal sampling quantity might be allowed. Recovery methods include sterile-swabbing (non-invasive nature) and use of tape (of a micro-invasive nature) according to the recent methodological development by Genova and collaborators (Genova et al., 2020), while other authors use sterile spatula for the removal of the first layer and collection of a section underneath (Ding et al., 2021). Minimally invasive methods are also important when considering other surfaces of biological origin as skeletal remains. These are key for analyzing among others ancient pathogen global dynamics or reconstructing the microbiomes (Farrer et al., 2021). In that context the issue is to recover high quality DNA while eliminating contaminating genetic material inputs. The removal of confounding genetic material can be achieved by surface decontamination (Meffray et al., 2019). For studies where sample amount was not as critical, swabbing can be applied as a suitable recovery method (Marotz et al., 2018).

A key aspect to consider, especially for low biomass samples, is the potential contamination with external DNA not related with the sample of interest (Eisenhofer et al., 2019). These contaminants could arise even from the commercial reagents used at isolation, and bias the reads obtained (Weyrich et al., 2019), thereby preventing rigorous analysis.

#### Overview on methods for extraction

An extensive and recent review on methods for nucleic acid extraction can be found in Emaus et al. (2020). Traditional liquid–liquid extraction methods often require large volumes of solvents as exemplified in a study to develop effective decontamination procedures when recovering dental microbial DNA in ancient human remains (Farrer et al., 2021). Thus, current developments revolve around the use of microfluidic devices allowing the use of volumes in the 10^−6^–10^−3^ l range (Vicente et al., 2020) and the subsequent exploration of a broad range of solvents such as magnetic ionic liquids that can be manipulated with external magnetic fields when used in complex matrices (Clark et al., 2015). Solid phase extraction methods limit the use of liquid solvents through replacement with sorbent solids (Price et al., 2009). Magnetic Solid-Phase Extraction combines a magnetic core to facilitate separation and this technique has been extended by the use of functionalized nanomaterials (Manousi et al., 2020). Graphene oxide nanocomposites are widely used, and DNA isolation techniques using these have been specifically developed for poor quality samples (Liu et al., 2019).

#### Extracellular DNA

Analysis of DNA in bacterial biofilms faces the presence of extracellular DNA in the extracellular matrix (Flemming et al., 2016). This external moiety of nucleic acids in the matrix might originate either from cell lysis or active lysis, and double or single stranded DNA fragments are found (Monticolo et al., 2020). DNA from lysed cells can be actively used as a nutrient and a number of bacterial species actively secrete extracellular nucleases to access these resources (Jakubovics et al., 2013). Actively secreted DNA through specialized mechanisms was already proved to be essential for biofilm formation 20 years ago (Whitchurch et al., 2002), and has been associated to many of the mechanical properties of biofilms (Gloag et al., 2020). Some authors have suggested that electrostatic interactions between eDNA (negatively charged) and secreted proteins (positively charged) contribute to the structural integrity of the extracellular matrix (Dengler et al., 2015; Kavanaugh et al., 2019). Mechanisms for bacterial DNA secretion are linked to quorum sensing systems, so that this process is tightly regulated during biofilm development (Ibáñez de Aldecoa et al., 2017).

Different views on microbiome composition might arise depending on the DNA source (intracellular or extracellular). A metagenomic analysis in deadwood (Probst et al., 2021) showed that community composition regarding most abundant organisms remained unaltered, however, the identity and relative abundance of less abundant variants was greatly affected by the origin of the DNA. Thus, methods for sequential extraction from extra- or intracellular compartments are required for each specific environment (Nagler et al., 2018), although the general principle is to extract extracellular DNA while preventing cell lysis, and for this task enzymatic methods are preferred over chemical ones (Wu and Xi, 2009).

#### Systematic optimization of extraction methods

DNA extraction for the study of microbial surface interactions in complex interfaces often requires the development of specific methods. Extraction methods need to be optimized for the problem at hand especially as they can greatly affect low biomass samples for microbiome analysis (Zoetendal et al., 2001), and negative controls are essential to deconvolute the effect of contaminations (Lauder et al., 2016). Pérez-Brocal et al. (2020) tested five DNA extraction protocols to analyze microbial communities using sequencing of 16S RNA (bacteria) and ITS (fungi) genes. A bead-beating step was seen to increase apparent abundance of Gram-positive bacteria such as Firmicutes and Actinobacteria with extensive peptidoglycan in their cell walls. Similarly, fungal families Malasseziacee and Aspergillaceae were more abundant in the corresponding samples. The use of negative controls allowed Pérez-Brocal et al. (2020) to avoid reporting taxa strongly and significantly overlapping with the negative controls. Optimization of methods and protocols often requires evaluation of numerous factors that might impact performance, and tools for statistical design of experiments can assist this process by minimizing the number of required experiments. Booncharoen et al. (2021) evaluated factors that can influence DNA extraction from bones such as the concentration of the chelator (EDTA), pH, incubation time and temperature, and volume of the solution. To fully explore the combined effect of all these factors, they used the Plackett-Burmann statistical method to design the experimental approach and the number of experiments for protocol optimization was reduced from 32 to 13, and enabled them to identify EDTA concentration as the major factor of success for the protocol (Booncharoen et al., 2021).

### Sequencing approaches: Technologies and platforms

Current sequencing platforms generate reads not long enough not to cover an entire microorganismal genome with a single read. For a long time, genomic and transcriptomic analysis have been dominated by second generation sequencing technologies, represented by Illumina technology (Illumina, Inc.), that deliver millions of high-quality short reads (150 ~ 500 bp). Third generation sequencing, illustrated by PacBio (Pacific Biosciences of California, Inc.) and Oxford Nanopore (Oxford Nanopore Technologies Limited) technologies, deliver reads of up to 10 kb. In earlier years, these technologies suffered from low accuracy, but this factor is continuously improving, partly due to the development of specialized software. Long reads have the advantage of allowing better resolution of genomic repeats or structural variation, although they remain less affordable than short read and have more strict requirements regarding sample quality. Protocols need to be adapted to ensure sufficiently long fragments. A summary of already existing protocols can be found in Maghini et al. (2021), still they might need further adaptation such as replacement of mechanical lysis by enzymatic lysis (Maghini et al., 2021). It should be noted that most currently available algorithms and bioinformatic analysis pipelines are more suitable for shorter reads, however more and more dedicated tools for analysis of long reads are emerging in this rapidly developing field (Amarasinghe et al., 2020). An excellent review on currently available sequencing technologies can be found in Hu et al. (2021).

Based on these sequencing technologies, two alternative approaches emerge to obtain complete genomes: *de novo* assembly and reference-based sequencing (also called resequencing). The latter uses already available genome sequences of type strains as a mapping template (Giani et al., 2019). The choice between either approach is dictated by the purpose of the research and most important by the availability of good references for the studied organisms. Currently, advances in sequencing technologies have delivered billions of nucleotide sequences that are available at publicly accessible databases, and new genome sequences are incorporated almost on a daily basis (Sayers et al., 2020; Harrison et al., 2021). In each project a number of steps, detailed in Dominguez Del Angel et al. (2018), have to be considered often in consultation with the sequencing facility. For example, the number of required sequences (sequencing depth) critically depends on estimated genome size and higher values are required for a *de novo* assembly. These requirements also vary with the structure of the genome, the sequencing technology, and the quality of the reads (Sims et al., 2014).

Advances in sequencing technologies (Ciuffreda et al., 2021), combined with the development of ultra-fast and memory efficient computational algorithms have enable genome-resolved metagenomics, that is the recovery of high-quality microbial genomes from complex environmental samples by *de novo* assembly (Kayani et al., 2021). For the analysis of microbial communities, in fact, longer reads from marker genes such as 16S rRNA or ITS enable better taxonomic resolution (Tedersoo et al., 2021). Similarly, longer reads can be used to complement short reads for finishing genome assembly and gap solving, as illustrated in seven bacterial genomes in Utturkar et al. (2017). An extensive review of all computational tools for generating metagenome-assembled genomes can be found in Gwak et al. (2021) and Yang et al. (2021) .

### Computational genomics

Once initial quality of the starting material has been guaranteed, genome analyses associated with interaction between microbial cells and natural or synthetic interfaces follow standard pipelines for genome assembly, and structural and functional annotation (Ejigu and Jung, 2020) or metagenomic binning, abundance analysis and functional interpretation (Bharti and Grimm, 2021). The main challenge in the study of microbial surface interaction is the functional annotation of relevant elements and pathways. Identification of genes and pathways specifically related to biofilm formation or the interaction with surfaces has recently been boosted by the potential of CRISPR for high-throughput functional genomics. Systematic disruption of gene expression levels using CRISPR interference has been used to interrogate control of biofilm formation in multiple strains of *Pseudomonas fluorescens* (Noirot-Gros et al., 2019). These can be complemented with computational tools. Protein location tools aim at predicting the subcellular location from the protein sequence through the recognition of sorting (or targeting) sequences and or the analysis of sequence features. Multiple tools are currently available and reviewed by Imai and Nakai (2020); these tools remain relevant even with the recent advances in proteomics technologies as they can offer whole genome coverage which is often not available for proteomics. Initially developed algorithms, such as p-sort (Nakai and Horton, 1999), target peptide signals, but algorithms were subsequently improved by including identification of transmembrane helices and extensive comparisons with proteins of known location. Currently, algorithms based on deep learning or other machine learning approaches exploit the wealth of available proteomics datasets for training. Still, no perfect method has been developed and most successful approaches are consensus methods combining predictions from different tools. Such is the case of the predictions in the BUSCA web server (Savojardo et al., 2018) that searches for protein features such as secretory or organelle-targeting peptides, GPI-anchors, and alpha or beta transmembrane regions by combining up to eight well established algorithms. It should be noted however, that in many cases membrane proteins are predicted based on transmembrane domains, best suited for integral membrane proteins. Membrane bound proteins have distinct features that makes their prediction elusive and dedicated algorithms for this task are currently developed, again combining prediction tools relying on machine learning approaches, such as TooT-M (Alballa and Butler, 2020). Moreover, we expect that recent breakthroughs on protein structure prediction will largely increase accuracy of location predictions based on sequence information (Jumper et al., 2021). Finally, the up-and-coming use of protein Language Models, basing their reconstruction solely on local and global sequence information, has also shown to improve the prediction tools considerably (Teufel et al., 2022).

## Generation and analysis of transcriptomics data for the study of microbial-surface interactions

Comprehensive mRNA sequencing is a powerful tool for conducting unbiased, quantitative differential gene expression analysis (LoCoco et al., 2020). However, the reliability of the obtained data depends on the extraction of high-quality RNA from samples. As in the case of DNA, the isolation of RNA with acceptable quality for RNA sequencing (RNA-seq) analysis from microorganisms present in macroscopic and microscopic, biotic and abiotic surfaces, is challenging, due to the presence of inhibitors, chelators, low amounts, or due to particularities of the experimental design, for example, when performing single cell transcriptomics analyses. Keeping RNA integrity during extraction can be problematic, especially in tissues such as skin with dense, connective matrices, and elevated ribonuclease expression. In addition, the ability of nanoforms to adsorb single-stranded nucleic acids is a burden for the isolation of RNA from cells that interact with them.

### RNA isolation from (nano)biotic/organic surfaces/matrices

#### Skin/animal tissues

Transcriptomics analyses of microbial strains interacting with animal biological tissues, such as skin and lung epithelial tissues, are becoming standard approaches to understand microbial lifestyles and pathogenicity (Guilhen et al., 2016). However, studying colonizing bacterial gene expression with RNA sequencing is challenging because the ratio of host RNA to bacterial RNA is very high in infected tissue which hampers obtaining enough bacterial reads to study bacterial expression levels. Methods for microbial RNA isolation and enrichment, tailored to the composition particularities of the multicellular matrix, have been published during the last years. For instance, a hybridization method, based on the coincidence cloning (CC) approach, for the isolation of representative bacterial cDNA pools from infected organs has been proposed as an easy-to-implement procedure. Co-denaturation and co-renaturation of the excess of bacterial genomic DNA with the cDNA transcribed from total RNA of the infected tissue enabled a >1,000-fold enrichment of a certain bacterial cDNA fraction (Azhikina et al., 2010). The use of commercial kits for the enrichment of microbial RNA from tissue samples has been described as well, in combination with specific methodologies to isolate bacterial fractions. In particular, the direct transcriptome analysis of *Mycoplasma hyopneumoniae* from pig tissue lungs was shown to be problematic due to the low bacterial mRNA available. However, the MICROBEnrich kit has been successfully employed in the isolation of enough bacterial mRNA for RNA sequencing, employing as a starting material infected lung flushes (Kamminga et al., 2020).

Skin also challenges the isolation of RNA from colonizing bacteria. For instance, the isolation of RNA from the pathogenic bacteria *Mycobacterium ulcerans* from skin tissue from a mouse model for RNA sequencing required the development of a method based on differential lysis (Robbe-Saule et al., 2017). First, the host cells were disrupted by a combination of tissue mechanical lysis and proteinase K treatment. This process does not have an impact on bacterial cells integrity, which can be separated from the lysate containing host RNA by centrifugation, and the pellet containing the bacteria is subsequently resuspended in lysis buffer, disrupted by bead-beating, and finally purified on a Zymo column.

The microbial biofilm matrix itself can significantly interfere during the RNA extraction. For instance, polysaccharides, the major component of the *Staphylococcus epidermidis* biofilm matrix (about 90%), a common inhabitant of normal human skin and mucosa, which has recently emerged as a leading cause of biofilm-related infections, make bacterial cell lysis and RNA purification difficult (França et al., 2011). França et al. (2011), compared different kits, with different characteristics, for RNA isolated quantity, purity, integrity, and functionally. The compared kits were: FastRNA^®^ Pro Blue (MPBiomedicals, Irvine, CA, United States), which employs mechanical and chemical lysis together with organic extraction; PureZOL™ RNA isolation reagent (Bio-Rad, Hercules, CA, United States), which uses chemical lysis with organic extraction; and PureLink™ RNA Mini Kit (Invitrogen, San Diego, CA, United States) that uses enzymatic lysis and silica membrane extraction. While all kits were able to extract intact and functional total RNA from *S. epidermidis* biofilms, the obtained results showed that the FastRNA^®^ Pro Blue kit was the only one able to isolate pure and large RNA amounts.

#### Plant tissues

Adapted RNA harvesting protocols have allowed *in vivo* transcriptomic analyses of microbes during interactions with their host plants. Studies focused mainly on legume/rhizobia systems, taking advantage of the high bacterial population present in nodules (Chapelle et al., 2015). However, analysis of colonizers in other plant tissues (e.g., leaves) has proven more challenging, due to the low proportion of their RNA in the host tissue samples (particularly in early colonization stages), and to the fact that plant RNA cannot be removed easily through bacterial RNA enrichment or eukaryotic RNA subtraction. Different methodologies have been described for bacterial RNA isolation from plant tissues, such as leaves and roots.

Chapelle et al. (2015) described a method for analysis of the gene expression of a bacterial pathogen at the initial stages of foliar infection. The method is based on bacterial cell isolation, through a density gradient centrifugation step of a disrupted and filtered plant tissue, which results in a 10^4^ to 10^5^-fold enrichment of bacterial RNA, compared to crude total RNA samples. To prevent changes during the process, the isolation is performed in the presence of an RNA stabilizing agent (Chapelle et al., 2015). More recently, Nobori et al. (2018) applied a similar protocol to obtain enough RNA from *Pseudomonas syringae* infected *Arabidopsis* leaves, to perform RNA sequencing analysis (Nobori et al., 2018). Dual RNA sequencing analysis, at a sequencing of >20 GB per mixed sample (bacteria infected plant leave), allows the *in vivo* transcriptional analysis of plant-bacteria interactions (Liao et al., 2019).

### RNA isolation from abiotic surfaces

During biofilm development, sessile cells acquire physiological characteristics differentiating them from planktonic cells. Increasing attention has been paid to gene expression levels, to understand surface sensing and biofilm formation. When initial responses to spontaneous cellular adhesion occur, only a limited number of cells are generally attached to the surface. Since different cells attach to the surface at distinct times, a significant heterogeneous population of cells is present and single-cell methods are very relevant at the initial stages (Kimkes and Heinemann, 2020). Continuous improvements of single cell isolation methods and RNA-seq technologies in relation to single cell sensitivity have enabled single cell transcriptomics analysis (Chen et al., 2017). For instance, Imdahl et al. (2020) have developed a protocol for cell sorting and RNA-seq analysis of bacteria, based on systematic cell isolation using fluorescence-activated flow cytometry (FACS) and MATQ-seq (Imdahl et al., 2020), a highly sensitive sequencing protocol able to detect transcriptional variation among cells of the same population (Sheng et al., 2017).

In regard to mature biofilms, the microbial cell physiology differs depending on their spatial location within the community, as gradients appear throughout the biofilm. To isolate different subpopulations of a bacterial strain within a biofilm, and study differences in gene expression that occur at localized sites and due to distinct environmental conditions, or stochastic gene expression events, laser capture microdissection has been used to isolate samples that can be subjected to transcriptomics analyses (Williamson et al., 2012).

RNA isolation for transcriptomics analysis from microorganisms present in abiotic surfaces can be challenging as well in many cases, due to the presence of inhibitors, chelators, low amounts, difficult access, etc. In case of bacteria, mRNA sequencing can be even more challenging, as it does not contain a poly-A tail at the 3′ end, which is commonly used to enrich these molecules during reverse transcription, considering as well that bacterial cells usually contain 100-fold lower RNA than mammalian cells, and more than 95% consists on ribosomal RNA (rRNA) (Wangsanuwat et al., 2020). To deal with these limitations, a number of solutions have been investigated. For instance, several commercial kits have been developed to remove bacterial rRNA from total RNA samples, such as MICROBExpress, RiboMinus and Ribo-Zero, which are based on subtractive hybridization to deplete rRNA. Another possibility is the specific degradation of rRNAs with 5′-monophosphate ends, but not mRNAs with 5′-triphosphate ends, employing Terminator™ 5′-phosphate-dependent exonuclease. Also, different technologies based on rRNA tiling and degradation are available. In addition, the Enrichment of mRNA by Blocked rRNA (EMBR-seq) technique has been recently introduced, based on the use of 5S, 16S and 23S rRNA blocking primers and poly-A tailing to specifically deplete rRNA and enrich mRNA during downstream amplifications (Wangsanuwat et al., 2020).

Total RNA chelation by active surfaces, such as certain nanomaterials, has been described when studying the global transcriptional response of microorganisms interacting with them, as well as the development of a specific methodology to overcome this issue. In particular, the obtention of high-quality total RNA in enough amounts to be used for RNA-seq analysis of yeast cells interacting with graphene oxide nanoparticles could only be achieved through the introduction of a separation step, prior to the start of the RNA isolation protocol (Laguna-Teno et al., 2020). The separation step, based on a sorbitol gradient separation process, allowed to separate yeast cells from graphene oxide nanoparticles, to successfully isolate ribonucleic acid levels (in concentration and purity) for RNA-seq analyses.

The successful purification of ancient RNA, including that from microbial origin, has been described, although not many studies are yet available. The most common case of recovery and analysis of ancient microbial RNA is that of viruses and viroids from ancient samples that had been preserved chemically, frozen or in a dry state (Guy, 2014; Smith et al., 2014). For instance, the RNA virus causing the 1918 great influenza could be isolated from formalin-fixed, paraffin-embedded, lung autopsy materials using proteinase K digestion, followed by either a phenol-chloroform or an acidic guanidinium thiocyanate-phenol chloroform extraction step, and isopropanol precipitation in the presence of glycogen (Krafft et al., 1997). The 1918 great influenza pandemic virus was also isolated from frozen, unfixed lung tissues, using RNAzol and manufacturer indications (Reid et al., 1999). Furthermore, by employing a viral particle-associated nucleic acid enrichment approach, DNA and RNA viral genomes from a 700-year-old fecal sample from caribou preserved in a subarctic ice patch were isolated and sequenced. Viroid RNA has been successfully amplified and sequenced as well from 50-year-old dried leaf material, using standard purification approaches (Guy, 2013). Few reports on the purification and sequencing of microbial RNA from archeological seeds have been published as well. Deep sequencing of ancient biological material, such as approximately 700 years old desiccated maize kernels, allowed to obtain microbial reads (Fordyce et al., 2013), including bacteria and archaea. In this study, nucleic acid extraction was performed using an optimized protocol, involving initial bleaching of the testa to minimize external contamination. Also, RNA purification and sequencing from approximately 750 years old barley grain allowed the reconstruction of the RNA genome from an ancient barley stripe mosaic virus (Smith et al., 2014). This required a modified protocol of the MirVana miRNA Isolation Kit, with extended (8 day) initial incubation of the sample in 1 ml CTAB buffer, following a standard isolation procedure.

### Sequencing approaches and computational analysis

Transcriptomics share the same sequencing technologies and platform of genomics. Dealing with microorganisms interacting with surfaces is also not entailing major adaptations in the data analysis workflow. However, the introduction of long reads sequencing has also pushed forward the transcriptomic field owing to the technology ability to produce near full or full-length mRNAs also without the need of reverse transcription (Ciuffreda et al., 2021). An example is the use of Oxford Nanopore in the direct detection of mRNA in a marine pelagic crustacean zooplankton community to monitor seasonal changes (Semmouri et al., 2020). In addition, microorganismal mRNA presents no or low percentage of introns in comparison to higher eukaryotes making the initial data treatment not very different from genomics. More challenging is the detection of transcripts in communities. The reason for the above lies once more in the still prominent use of Illumina sequencing technology that generates high-throughput short RNA-seq reads. However, many tools have been developed to tackle this problem as reviewed by Shakya et al. (2019).

When analyzing transcriptomics data, the interest is on differences in gene expression. These can indicate what is actively transcribed in the condition under consideration and give insights on the specific functions possibly happening in the different conditions. Many tools have been developed to normalize and calculate (with the most widely used being the R packages limma, EdgeR and DeSeq2) and their performance and usability is continuously assessed as for example in Corchete et al. (2020). Mapping back the transcripts to related genes enables the use of both KEGG (Kyoto Encyclopedia of Genes and Genomes) pathway mapper and Gene Ontology enrichment covering biological process, molecular function, and cellular component to gain more biological information. These tools allow the retrieval of cellular, molecular and organismal-based functions that are enriched in the up or down regulated samples as exemplified in Laguna-Teno et al. (2020). Same analysis pipeline, yet with dedicated software tools, can be applied to the study of an entire community of microorganisms with the aim of identifying the presence of collective functionalities. In such a metatranscriptomics approach the information on genes that are expressed in the community can tell whether for instance a resistance gene is actively transcribed or the microorganismal individuals are viable. Of particular interest is the possibility of using Oxford Nanopore RNA-sequencing *in loco* to easily detect the above mentioned characteristics in food or clinical samples as reviewed in Ciuffreda et al. (2021). Software tools for metatranscriptomics analysis are still at their infancy and therefore in continuous development. For an analysis of present tools, current challenges and promises of metatranscriptomics analysis we refer the reader to Shakya et al. (2019) with attention on current best practice presented in Chung et al. (2021).

## Generation and analysis of proteomics data for the study of microbial-surface interactions

With the development of more sensitive mass spectrometry (MS) instruments and quantitative approaches, proteomics has become a routine analysis for investigating the protein molecular effectors within any cell type. The proteome, or else the ensemble of the proteins present in the analyzed sample at a given time, can be easily recorded by MS for many types of live specimen (Mann et al., 2013).

Cell-surface and cell–cell interactions of microorganisms are physically mediated among others by proteins. Their study represents a unique opportunity to understand how these microorganisms can colonize biotic and abiotic surfaces to their advantage. Proteomics offers the additional benefit of being able to dissect multiple levels of the interaction’s players. In fact, various methods have been developed to extract the proteins released by the cells in the extracellular environment (secretome), to probe for proteins directly exposed in the surface of the cells (surfaceome), and finally to look at intracellular proteins globally or at the level of specific organelles. Collecting this multi-layer information is hampered by the quantity of starting material. In addition, separating the microbial component from the surface or interacting biotic material is not always trivial as well as producing a clean sample. However, the downstream processing of the protein sample follows the standard pipeline of cysteine reduction and alkylation, enzymatic digestion into peptides, peptide desalting and concentration, and finally MS analysis of the peptides. An additional barrier to the successful identification and quantification of cell-surface and cell–cell protein interactions is represented by the data analysis that normally requires *a priori* genomic information and annotation, and that suffers from the sparsity of collected data.

### Protein extraction from biotic surfaces

#### Animal tissues

The analysis of host-associated microbiome samples presents several challenges related to the efficient and unbiased protein extraction. Differences in the cell envelope between Gram positive and negative bacteria and the presence of extracellular microbial proteins affect sample preparation. Evaluations of multiple extraction methods are therefore important. Such an approach sets the use of SDS and ultrasonication as the best method to date for human gut metaproteomic sample preparation (Zhang et al., 2018). Another challenge is posed by contamination due to the presence of a large proportion of host proteins overtaking the microbial ones, and the presence of contaminants such as food residues. Starr et al. (2018) recently presented an extensive review of proteomics and metaproteomic approaches developed to study host microbe interactions.

Interactions between the host and the microorganisms colonizing it can be collected from several compartments of the body for example saliva and plaque, intestinal mucosa, skin, bronchoalveolar lavage fluids (BALF). In the metaproteomic field, samples for the analysis of gastrointestinal tract prevail over other types of samples because the mucosa represents the second greatest interaction site between the microbes and their host, and the most richly populated microbial surface. Extraction methods for the gut microbiome are also focused on combining multiple omics technologies. Keller and colleagues investigated three different extraction methods for the analysis of metabolites, peptides, and proteins using mouse cecum as a sample. To detect the largest number of small molecules and identify the largest number of peptides, metabolomic and peptidomic samples are best prepared by methanol/chloroform/water extraction. However, the highest number of protein identifications was obtained with the acidified methanol extraction for proteomics sample preparation. The combination of multiple omics technologies asks for a compromise in the extraction methods more suitable to specific omics technologies (Keller et al., 2021).

Microbial lung infections are less studied by metaproteomics. However, respiratory diseases caused by microbes can be investigated not only *via* a biopsy of lung tissue but also using expectorated sputum and BALF. Invasive pulmonary aspergillosis caused by *A. fumigatus* was recently studied in human BALF and mouse models using extraction of proteins *via* eFASP (Erde et al., 2014). This analysis detected candidate biomarkers for the infection (Machata et al., 2020). Cystic fibrosis is a genetic disease in which pathogens play a major role in the disease progression. To study the pathophysiology of the microbial community in cystic fibrosis, recently protocols to better purify the microbial pathogens from sputum were developed based on differential centrifugation and detergent treatment to enrich bacterial cells. The extraction method improved the identification of the proteome from sputum of *Pseudomonas aeruginosa* infected cystic fibrosis samples (Wu et al., 2019). A more recent version of such protocol is based on mechanical homogenization combined with extracellular DNA aggregates digestion by DNAase I and differential centrifugation steps for the enrichment of microbial cells. The metaproteomics analysis identified enriched proteins from various bacterial genera and highlighted that arginine deiminase pathway and multiple proteases could be contributors to the pathophysiology of cystic fibrosis (Graf et al., 2021).

In the analysis of whole proteomes, a new field is represented by paleoproteomics the analysis of ancient proteins. Owing to the superior biomolecular preservation of proteins in comparison to DNA, paleoproteomics has the potential to go further back in time. It relies on samples that are less prone to contamination with recent molecules in comparison to DNA. Since no amplification step is necessary causing extra contamination, the amount of protein material recovered becomes more binding (Buckley, 2019). Most of the research in the past years has been focused on the analysis of biomineralized ancient tissues namely bones and teeth. For example, protein signatures left by microbes in dental pulp samples can be used to diagnose ancient infectious diseases. Proof of principle was the identification of proteins from *Yersinia pestis* in 300 years-old dental pulp collected in plague-positive sites in France (Barbieri et al., 2017). Analysis of ancient proteins present on archeological artifacts (Vilanova and Porcar, 2020) or remains can also highlight the presence of ancient infections. Greco et al. (2018) managed to purify using the PlusOne 2-D Clean-Up kit (GE Healthcare Life Sciences) proteins from ancient Egyptian cheese and identify the presence of *Brucella melitensis* and date a brucellosis infection to the Ramesside period (Greco et al., 2018).

#### Ground and plants

Protein enrichment from soil and litter samples is obtained by aqueous buffers in combination with mechanophysical treatments, use of organic solvents, detergents, and strong acids or bases (Keiblinger and Riedel, 2018). Soil and litter protein analysis is challenged by the broad heterogeneity of the samples with respect to characteristics such as electrical conductivity, texture, pH, carbon–nitrogen–phosphorus stoichiometry or humic acid content (Starke et al., 2019). For example, humic compounds adsorb proteins and represent an obstacle to the extraction, purification, and quantification of proteins but can be mostly removed *via* acid filtration (10 kDa) (Qian and Hettich, 2017). A combination of extraction strategies is recommended to achieve higher coverage in the metaproteome samples. This could include SDS–phenol and TCA precipitation or NaOH extraction methods (Chourey and Hettich, 2018). SDS-based protocols are indicated for efficient identification of proteins from soil (Bastida et al., 2014) while NaOH is suitable to extract specific proteins covalently bound to clay particles.

Microbially-driven soil processes are responsible for plant organic matter mineralization and stabilization. An example study looked at the lignocellulose decomposition happening *in situ* within the surface level sediments collected from a natural established salt marsh located in the United Kingdom. Lignocellulose is permeated with the phenolic heteropolymer lignin that makes lignocellulose more hydrophobic and resistant to enzymatic degradation (Leadbeater et al., 2021). To look at the extracellular (secretome) and transmembrane (surfaceome) proteins, crosslinking of ε-amino groups of lysine with NHS-ester biotinylation reagents (i.e., Sulfo-NHS-SS-Biotin) was used. This method can label the cell surface primary amine of proteins without affecting the membrane and thus reducing cytoplasmic contamination (Hurley et al., 1985; Bonn et al., 2018). Extraction with SDS and protein precipitation are then used (Alessi et al., 2017). The label allows for the purification of the surface proteins *via* affinity with streptavidin and subsequent elution thanks to the cleavable arm of the cross-linker reagent. Finally, proteins can be identified *via* standard liquid chromatography and mass spectrometry (LC-MS/MS) pipelines.

To detect the secretome of *Hypocrea jecorina* fungus growing on bagasse, birch or spruce wood, or pure cellulose a new culturing method was created using agar plates containing the insoluble substrates and a low protein binding hydrophilic polyethersulfone membrane. In this way it was possible to collect the diffused secreted enzymes on an area under the membrane, thus avoiding fungal cell contamination (Bengtsson et al., 2016).

Plant proteomes can be profoundly affected by pathogens and beneficial microbes as recently reviewed (Jain et al., 2021). Efficient separation of plant, bacterial and fungal surface proteins from barley grains could be achieved with 25 mM sodium acetate pH 5.0 containing 0.02% (w/v) sodium azide, followed by centrifugation and filtration of extracted proteins (Sultan et al., 2016). Stable isotope probing approach can be used to track the nutrient flows from isotopically labeled substrates to certain microorganisms in microbial communities. This tracking of carbon flows from ^13^CO_2_ to the rhizosphere communities was performed in *Zea mays*, *Triticum aestivum*, and *Arabidopsis thaliana*. Rhizosphere soil was harvested from roots by separation from the loose soil and transferred in a harvesting buffer. The solution was then filtered (100 μm nylon mesh cell strainer) and centrifuged to obtain a pellet. A soil pellet was prepared by grounding it to fine powder in liquid nitrogen and a commercial kit NoviPure Soil Protein Extraction Kit (Mo Bio Laboratories) was used to extract proteins from soil and streamline the extraction (Li et al., 2019). The same kit was used for the release of proteins from the rhizosphere of *Vitis vinifera* for metaproteomics analysis (Bona et al., 2019).

### Protein extraction from abiotic surfaces

Interactions of microorganisms with abiotic surfaces involve among others nanomaterials, and biomaterials. To investigate how microorganisms react to microgravity environments and understand the exposure risk for humans in closed environments, particulates scraped from HEPA filters and from polystyrene wipes sampling the cupola surfaces of the international space station were analyzed. In this case, samples were first resuspended in sterile phosphate-buffered saline (PBS; pH 7.4), concentrated on a 0.45-μm filter and plated on two different media (R2A and PDA) to allow the growth of both environmental bacteria and fungi. Two *Aspergillus fumigatus* strains were identified and characterized by proteomics showing an increase in stress responses, and carbohydrate and secondary metabolism proteins (Knox et al., 2016; Blachowicz et al., 2019).

When focusing on the analysis of bacteria interacting with abiotic surfaces, proteomics can shed lights not only into the formation of sessile microbial cells organized in biofilms (Sauer, 2003) but also in the global changes arising by the altered molecular physiology of the bacterial cells when engaged in a biofilm (Ram et al., 2005). To investigate the formation of sessile microbial cells, cell surface biochemical components representing the surfacesome, also called surfaceome or surfome, of bacteria can be purified and analyzed. These proteins are key for the adhesion of the cells to inert surfaces and to each other.

Proteomics samples can be prepared from biofilm and bacteria entrapped in polymeric scaffolds (i.e., hydrogel) that mimic biofilm condition *via* conventional sample preparation and quantitative MS methods. For example, *Shewanella oneidensis* changes in protein expression were recorded in case of growth in an alginate hydrogel and biofilm by iTRAQ labeling (Zhang et al., 2014). Cells were resuspended in PBS and lysed in 0.1% SDS, 0.5 M TEAB by vortexing and ultrasonication. Newer isobaric labeling tags such as TMT are also routinely employed. For example, TMT labeling was used to understand the effect of silver nanoparticles on *Pseudomonas aeruginosa* biofilm where 3,672 proteins were identified and >600 showed a significant change (Liao et al., 2019).

Proteomics can also shed light into the attachment of microbes and formation of biofilms on biomaterials such as metals, alloys, ceramics, and polymers. Widely studied cases are orthopedic and dental implant devices. New coatings are continuously being developed to inhibit bacterial growth and biofilm formation on those devices (Arciola et al., 2018). It is therefore important to study how bacteria adhere to the biomaterials. For example, bacterial adherence proteins were extracted from the surface of *Staphylococcus epidermidis* after directly adhering to titanium implant materials. The bacteria were resuspended in osmotic lysis buffer with 20% sucrose (*w*/*v*) in 20 mM Tris–HCl, pH 7.0, supplemented with 10 mM MgCl_2_, and a protease inhibitor cocktail and treated with 100 U/ml of mutanolysin enzyme for 1–2 h at 37°C. Subsequent centrifugation removed the intact protoplasts. This allowed the identification of 6 relevant proteins on the surface of *S. epidermidis* (Bürgers et al., 2018).

### Proteomics sample preparation

While the perfect sample preparation relies on *ad hoc* separation and protein extraction methods, any improvement in the standardization of the bottom-up proteomics pipeline will also benefit the analysis of cell–cell and cell-surface microbes’ interactions by proteomics. Increasing the throughput and reproducibility as well as scaling down the proteomics workflow are aspects to consider. An example of the improvement in throughput is the “cell-to-peptides” workflow developed for fungi and Gram-negative bacteria using an integrated and automated platform. This includes equipment that allows for the cell lysis, protein precipitation and so removal of metabolites and lipids, subsequent protein resuspension and normalization, followed by tryptic digestion. Through the protocol 96–384 samples can be prepared in a few hours in a highly reproducible manner (Chen et al., 2019).

Gram-negative *L. pneumophila*, Gram-positive *S. aureus*, and a capsuled Gram-positive *S. suis* were used as test samples for the development of a “universal” protocol for bacteria available in limited numbers (2 × 10^6^ bacterial cells per sample corresponding to 0.12–0.9 μg protein per sample). Use of detergent (10% SDS) and ultrasonication disruption are combined with the beads-based single pot solid-phase enhanced sample preparation (SP3) method that allows for efficient removal of debris, detergents, and salts.

This protocol opens the doors to the use of samples from animal experiments, human origin, and the environment (Blankenburg et al., 2019). SP3 can also be automated (autoSP3) using a liquid handling robot to care for the preparation of 96 samples at the time (Müller et al., 2020) bringing together miniaturization and high throughput.

### Computational proteomics

When considering computational methods for the analysis of proteomics data in the context of cell–cell and cell-surface analysis of microorganisms, pipelines use a traditional approach of MS/MS peptide spectral matching based on database search. However, availability of a reference proteome is key to this database search strategy which can be limited due to the complex composition of microbial samples. Therefore, MS-based proteomics of microorganisms has grown hand in hand with the availability of the respective genomics information. Genomic and transcriptomic data can be used to generate *ad hoc* protein sequence databases to aid the interpretation of proteomic data in what is called a proteogenomic approach. In turn, the proteomic data deliver protein-level evidence of the gene expression data and support the refinement of gene models. Finally, the enhanced gene models can help improve protein sequence databases for traditional proteomic analysis (Nesvizhskii, 2014).

More complex samples and the need for larger reference databases represent bigger computational challenges for metaproteomics analysis compared to pure culture proteomics. Recently, open-source software solutions such as MetaProteomeAnalyzer and Prophane are making metaproteomics data analysis more at hand. Their advantages stand in combining different search engines for the peptide-spectrum and using a more comprehensive set of available databases (such as NCBI, UniProt, EggNOG, PFAM, and CAZy) for improved annotation (Schiebenhoefer et al., 2020). The database used for the metaproteomics analysis has a large influence on the results obtained as recently reviewed (Blakeley-Ruiz and Kleiner, 2022).

## Generation and analysis of metabolomics data for the study of microbial-surface interactions

Metabolites, compounds usually below 1.5 kDa of molecular weight, are often products of a variety of biological processes and reactions (Roberts et al., 2012; Zhang et al., 2018; Jacyna et al., 2019). They are a fine molecular reflection of the phenotype, as they depend on the specific conditions of the cells and can even differentiate between a wild type and a mutant specimen (Fiehn et al., 2000). Metabolomics provides a quantifiable layer of confirmation from the observed molecular events (Kanani et al., 2008). Measurements of metabolite concentrations along active pathways have largely been used for optimizing biotechnological processes, such as fermentation (Khakimov et al., 2017). This omic approach allows to determine accurately the levels of metabolites at each growth stage, and facilitate the optimization of production (Zhao et al., 2019) and it can help with screening for genetically improved strains (Liang et al., 2020).

Primary metabolites are associated with basic processes of production or breakdown of substrates (Demain, 1980) while secondary metabolites are linked to “social” interactions such as coordination or competition with other bacterial strains (Tyc Olaf et al., 2017). These analytes can belong to the intracellular or extracellular moiety and secreted metabolites can mediate communication and coordination between organisms not only in highly populated structures, such as the quorum sensing mediated by metabolites in biofilms (Rodrigues and Černáková, 2020), but also during interkingdom communication (Thaiss et al., 2016).

There are two main types of metabolomics instruments or platforms: nuclear magnetic resonance (NMR) and mass spectrometry (MS) (Sugimoto, 2021). NMR has a non-invasive nature, meaning that the sample does not have to be sacrificed. It allows for applications such as real-time analysis of metabolite fluxes, requiring no treatment of samples. It is suitable for highly polar compounds, and it is not restricted to fluids (Emwas et al., 2019). It has clear drawbacks: a limited number of detected metabolites due to lower sensitivity, spectral overlaps, very limited databases, a lack of bidimensional NMR data generation, or elevated technical costs related to maintenance and purchase of the instruments (Wishart, 2019). MS can be mainly divided into gas chromatography coupled to MS (GC–MS), and liquid chromatography coupled with single and double stage MS (LC–MS or MS/MS) (Zeki et al., 2020). GC–MS requires a low amount of samples, due to high sensitivity. The databases for compound identification in untargeted mode are well developed, since this technology has been in use from the early 2000s (Kanani et al., 2008). One of the main disadvantages in GC–MS is that samples have to be derivatized prior to the run, with the exception of volatile or non-polar analytes (Moros et al., 2017; Zeki et al., 2020). LC–MS offers a higher resolution than GC–MS, without the need for derivatization, and detecting a wide range of analytes (Favre et al., 2017; Zeki et al., 2020). Semi-polar metabolites are preferentially analyzed in this platform (Zeki et al., 2020).

### Methods for isolation of metabolomes

Two main approaches can be distinguished: targeted (analysis of a predefined set of metabolites) and untargeted (analysis of the whole set of metabolites according to instrumental capabilities) metabolomics. However, sample preparation including recovery, quenching, and metabolite extraction, tend to be similar for both targeted and untargeted metabolomics. Differences in protocols appear at the biofilm recovery stage, which varies according to the studied material. In addition, a deproteinization step is often carried out in the targeted approach (Iwasaki et al., 2012).

Metabolomic samples require the material to be treated quickly and at low temperatures to prevent changes that may be triggered by cell lysis (Zhang and Powers, 2012). Procedures that include flash freezing or lyophilization of samples aim to provide a snap-shot of the metabolite profiles, through enzymatic quenching, which has the objective of stopping the metabolism at a certain condition or treatment without further modifications (Teng et al., 2009; van Gulik, 2010). Low temperature processing and storage increase the stability of metabolites contained in biological fluids for longer periods and enhance potential for quantification (Scalbert et al., 2009; Kok et al., 2019). Sample preparation also has the objective of enriching some metabolites of interest (especially in targeted metabolomics), while removing substances that may bias the analyses (Gika and Theodoridis, 2011; Gong et al., 2017).

In the case of a biofilm, sampling usually begins at a washing step intended for removing growth media and loosely attached planktonic cells that could contribute with confounding signal (Sadiq et al., 2020; Guo et al., 2021). In addition, biofilms have to be detached first due to their recalcitrant nature. This represents several issues that depend largely on the type of surface.

#### Biotic surfaces

Stringent detachment methods cannot be applied to biotic surfaces without mixing the two metabolic profiles. The high specificity of metabolic profiles is exemplified even when comparing the molecular behavior of bacteria in biofilm or planktonic stages (Lu et al., 2019). Enzymatic treatments targeted to the biofilm can allow the recovery of the biomass without disruption of host tissues. However, the enzymatic treatment has a clear drawback due to incubation at room temperature leading to alteration or degradation of metabolites (Stipetic et al., 2016). Alternative methods include sonication treatment aimed at bacteria inhabiting the root rhizosphere (Noirot-Gros et al., 2018). Other studies use mechanical methods such as bronchoalveolar lavage to study lung samples from infected mice (Tomlinson et al., 2021). Harsher mechanical disruption was employed to study bacterial samples grown on autoclaved chicken meat (Dupre et al., 2019). In this case, the detachment was attained by disruption of the meat tissue with a pipette and using PBS as the homogenization solution. A first very low speed centrifugation step was intended to eliminate cell debris (mainly the disrupted meat substrate), and a second centrifugation for pelleting the cells of interest (bacteria). A similar approach using first a full matrix homogenization in mortar and pestle in the presence of liquid nitrogen was applied to study the surface cheese metabolome (Afshari et al., 2020).

Paix and collaborators (Paix et al., 2020) used a methanol bath to recover the surface microbial metabolome, similarly to the approach used by Guo et al. in where leaves were gently suspended in the presence of methanol, with further centrifugation and derivatization of the resulting supernatants (Guo et al., 2018). Recovery of surface metabolites can also be achieved with a solid phase material, as demonstrated with the surface metabolome of *Fucus vesiculosus* (Cirri et al., 2016; Parrot et al., 2019). Lastly, due to ethical constraints related to research with human subjects, the surface skin metabolome is sampled using adhesive tape and quenching is achieved by immediate storage at ultralow temperatures (Roux et al., 2021).

In between a biotic and an abiotic surface stands the enamel, namely the outer human tooth layer, which is composed mainly of 95% carbonated hydroxyapatite (Robinson et al., 1998), and develops through mineralization of a proteinaceous matrix (Gil-Bona et al., 2020). da Costa Rosa et al. (2021) induced dental caries lesions over the premolars of healthy individuals. The biofilm grown on appliances of extracted teeth were dried and removed with a phosphate/sodium azide buffer, or those at occlusal surfaces through a microbrush appliance.

#### Abiotic surfaces

Mechanical detachment from abiotic surfaces can be achieved through inert scraping tools to recover most of the biomass from polystyrene surfaces (Sadiq et al., 2020). Alternatively, the experimental design can include a direct step of cell disruption with glass beads in the presence of a solvent solution chloroform:methanol:ddH_2_O (Stipetic et al., 2016) aiming to quench metabolism. In comparison with scraping, it presents the advantage of congealing the metabolism at the exact moment when the cells are broken, because all metabolites are immediately released, and enzymatic reactions quenched. Another approach consisting of lyophilization of biofilms attached to metal coupons was used in combination with exposure to 50% methanol and use of vortexing as a mechanical disruption step (Pu et al., 2021). Other methods use a step of ultrasonic treatment prior to detachment (Zhao et al., 2019). Lastly, alternative approaches have successfully applied other chromatographic techniques without the need of scrapping as demonstrated by Brauer et al. (2017), or even without the need to disrupt the sample at all, such as a targeted assay where volatile metabolites were continuously screened (Slade et al., 2019). Finally, the biomass recovery issue can be solved also by pooling together several technical replicates that can increase the quantity of available material and aid to overcome the potential loss of material during sampling.

### Metabolite extraction

The selected extraction solvent depends on the platform to be used but it affects the type of metabolites that can be recovered (Cheng et al., 2020). The highest coverage is achieved with a combination of solvents, at the expense of a significant increase in labor and costs. Examples include pure methanol, 50% methanol (Pu et al., 2021), ice-cold chloroform:methanol:ddH_2_O (1:3:1) (Stipetic et al., 2016; Weidt et al., 2016), methanol:H_2_O:chloroform (2.5: 1: 1) (Afshari et al., 2020), acetonitrile, a mixture of equal volumes of acetonitrile and K_2_HPO_4_ - NaH_2_PO_4_ buffer (0.1 M, pH 7.4) (Zhao et al., 2019), or combinations of acetonitrile with chloroform.

Besides the bias that every solvent introduces, it is also important to consider that some types of metabolites can be affected if the solvent has not been adapted for that specific purpose. In that regard, Rabinowitz and Kimball (Rabinowitz and Kimball, 2007) developed an acidic acetonitrile/methanol/water mixture that largely avoided the decomposition of triphosphate intracellular components. Other extraction procedures use a hot methanol approach at 70°C instead of the usual low-temperatures (Yu et al., 2018). While it has been stated that cold conditions are needed, these are more important at the quenching step than in the metabolite isolation. Cell debris is precipitated by centrifugation and supernatants containing the metabolite extracts can further be mixed with acetonitrile for protein precipitation (Polson et al., 2003), prior to another step of centrifugation. Protein precipitation is an important step due to improved spectral resolution (Zhang et al., 2018). Supernatants are filtered and can be then lyophilized for long-term storage. Removal of methanol or acetonitrile is usually carried out by vacuum or by the mentioned lyophilization (Favre et al., 2018; Lu et al., 2019). Interestingly, these last two studies have also coupled quenching and metabolite extraction.

When considering sample preparation for GC–MS, samples are subjected to derivatization for the analysis of metabolites that contain polar groups and are not volatile (Moros et al., 2017). This procedure also offers advantages in terms of accuracy, sensitivity, or range of detection among others in LC–MS platforms (Zhao and Li, 2020). The derivatization is very specific to the targeted chemical groups (amines, phenols, thiols, etc.) and the matrix (Ollinik et al., 2021), and once added to the samples, the reagents and specific metabolites react to yield the derivatized compound (Zhao and Li, 2020).

### Computational metabolomics

Regarding computational approaches no differences in the pipelines can be identified when specifically analyzing cell-surface interactions. The massive information flow in the age of big data has pushed the development of suites and software platforms intended for data processing, annotation, and provision of a biological meaning to raw signals originating from the instruments. Many of these tools have been developed in the R environment, but still require an effort of bioinformatic adaptation and learning from the would-be users. Issues often arise due to non-standardized analysis pipelines, and the great variety of available software.

Upon completion of the run, a set of peaks or features are available for the user (Mahieu et al., 2016). For both MS platforms, the data includes intensity, mass (*m/z*), and retention time (Yao et al., 2019). These peaks have to be divided between real signals and noise (Styczynski et al., 2007). There are several platforms for data analysis of MS data that allow carrying out peak detection and alignment (Gardinassi et al., 2017; Tsugawa, 2018). Among them are MS-DIAL (Tsugawa et al., 2015), MZmine (Pluskal et al., 2010), XCMS (Mahieu et al., 2016), OpenMS (Röst et al., 2016), ADAP (Jiang et al., 2010), apCLMS (Yu et al., 2009), or MAVEN (Clasquin et al., 2012; Table 1). Recently, for the R XCMS software, a detailed protocol has been published (Yao et al., 2019). The databases of choice in that specific case for GC–MS were the GOLM metabolome retention indexed spectral library (Kopka et al., 2005), and NIST Main EI MS Library (NIST SDR 1A v14/v17). Once the peaks are identified, correspondence to the same analyte in different runs in the platform has to be established. The main cause of failure at this step is that the same analyte can display both different retention time and m/z, as illustrated by Mahieu and colleagues (Mahieu et al., 2016). As the practices are diverse, relevant cited examples have been organized in Table 1. This table reflects the diversity in computational approaches for metabolomics data interpretation in the study of cell-surface interactions.

**Table 1:** Computational approaches used in cell-surface interaction research studies.

Ref.	Bioinformatic analysis	Database	Statistics
Stipetic et al. (2016)	XCMS Smith et al. (2006) MzMatch and PeakML Scheltema et al. (2011)	Set of standards Match to literature Sumner et al. (2014)	Bayes moderated *t*-tests Smyth (2004) FDR PCA
Favre et al. (2018)	Data Analysis (v. 4.3, Bruker Daltonics) XCMS Simca 13.0.3 in-house scripts	XCMS DB	PCA PLS-DA
Dupre et al. (2019)	HP Chemstation (Agilent) AMDIS (NIST)	NIST02 (National Institute of Standards and Technology, Gaithersburg, MD, United States) WILEY7n (Palisade Corporation, Ithaca, NY, United States) Custom libraries	Student’s t-test PCA
Lu et al. (2019)	GC–MS: XCMS and AMDIS LC–MS: AB SCIEX Analyst	XCMS DB (GC–MS)	MetaboAnalyst 3.0: multivariate analysis
Slade et al. (2019)	MATLAB R2018a	N/A	Time course mean
Zhao et al. (2019)	TopSpin 4.0.3 (Bruker, Rheinstetten, Germany) Amix package (version 3.9.15, Bruker)	BMRB ECMDB	SIMCA-P+, v. 11.0: multivariate analysis MetaboAnalyst 4.0
Paix et al. (2020)	LC–MS: Data Analysis (v. 4.3; Bruker, Germany) XCMS Workflow4Metabolomics (W4M) in Galaxy environment Giacomoni et al. (2015) GC–MS: MSD ChemStation (v. F.01.00.1903) R package “eRah” Domingo-Almenara et al. (2016)	Comparison with standards Wiley 2008 NIST 2011	R MetaboAnalyst: Multivariate analysis PCA, PLS-DA
Sadiq et al. (2020)	Shimadzu GCMS PostRun MS DIAL Origin (V)	FiehnLib	PCA PLS-DA MetaboAnalyst
da Costa Rosa et al. (2021)	Topspin (Bruker Biospin) AMIX programs (Bruker Biospin, Germany) Metaboanalyst 2.0	Human Metabolome database	PLS-DA O-PLS-DA
Guo et al. (2021)	Agilent proprietary software	Comparison with standards	GraphPad Prism 6.0 Microsoft Office (Excel 2013) MetaboAnalyst v. 4.0
Pu et al. (2021)	ProteoWizard Kessner et al. (2008) XCMS Smith et al. (2006) CAMERA Kuhl et al. (2012) MetaX Wen et al. (2017) Rstudio (version 3.6.0)	HDMB Wishart et al. (2013) MassBank Horai et al. (2010) Lipidblast Kind et al. (2013)	Student’s *t*-test
Tang et al. (2021)	XCMS	MetDDA LipDDA (*In-house databases*)	PCA OPLS-DA
Tomlinson et al. (2021)	E-Maven v0.10.0. Clasquin et al. (2012); Agrawal et al. (2019)	Comparison with standards	Two-Way ANOVA with Dunnett’s Multiple Comparisons

BMRB, Biological Magnetic Resonance Data Bank; ECMDB, the *E. coli* Metabolome Database; FDR, false discovery rate (Röst et al., 2016); PCA, principal components analysis; PLS-DA, partial least squares-discriminant analysis; NIST, National Institute of Standards and Technology; OPLS-DA, orthogonal partial least squares-discriminant analysis.

Within some of the examples used from biofilm research, Favre et al. (2018) used XCMS for preprocessing of their untargeted metabolomics acquired data, including suppression of redundant signals (additional script provided by authors). At the stage of data analysis, discrimination was performed through principal component analysis (PCA) and partial least-square discriminant analysis (PLS-DA). This statistical analysis seems to be widely used (Table 1). The variable importance in projection (VIP) concept was termed, highlighting the highest contribution of analytes to their biomarker selection model. The VIP value was used for selection of the metabolites to annotate, relying on public databases: Metlin^1^, KEGG^2^, Pubchem (Kim et al., 2016), Chemspider^3^, and Lipidmaps^4^. These last tools highlight the importance of visualization in order to obtain a general view of the metabolism and be able to draw relevant biological conclusions out of a vast amount of data. Tang and colleagues (Tang et al., 2021) used a similar data analysis approach (XCMS), yet employing in-house built MetDDA and LipDDA as reference databases (not publicly available).

In their targeted metabolomics approach, Guo et al. (2021) used the proprietary Agilent software (Agilent Technologies, United States) for peak processing. Adopting Proprietary software for the conversion of raw data into processable information is another common practice. Further normalization was achieved using the experimental values of colony-forming units (CFU), where the normalized values were processed with MetaboAnalyst (Pang et al., 2021). This tool can be coupled into the OmicsAnalyst, to integrate several omics data (Zhou et al., 2021).

An internal standard can serve for comparison between samples (Dupre et al., 2019). In their study, GC–MS data was compared with the libraries (National Institute of Standards and Technology, Gaithersburg, MD, United States), WILEY7n (Palisade Corporation, Ithaca, NY, United States), while for analysis of chromatograms and spectra they used HP Chemstation (Agilent Technologies, United States) and AMDIS (NIST, MD, United States).

## Integration between omics datasets

When coombining data generated by different omics, we get closer to a factual representation of the molecular mechanisms responsible for a certain phenotype. For example, an extensive multi-omics approach involving meta-genomics and -proteomics, and metabolomics was used to study the microbial structure, enzymatic repertoire, functional traits, and relevant metabolic pathways of a pre-denitrification biofilter installed in a WWTP in an urban area in China (Tian and Wang, 2021). Recently Li and Wen (2021) highlighted how multi-omics approaches have a prime role in the bio-mining of microorganisms from extreme environments and the understanding of how they dissolve metal-sulfide elements (bio-leaching) or break down the mineral matrix (bio-oxidation).

Statistical analysis methods such as PCA or PLS are commonly used for multiple omics techniques to reduce the dimensionality of the data and evaluate the similarity between samples from (biological) replicates. In that regard, we have highlighted some of the statistical approaches used by several studies included within this review (Table 2). In addition to these similarities in the statistical treatment, a commonly discussed outcome of omics technologies is the reconstruction of metabolic pathways and provision of functional annotation. The issue, especially with genomics and to a lesser degree with transcriptomics, is that we are not always certain of the downstream events. If we consider the number of “features” obtained by each omics technology, we observe a trend toward reduction of obtained features when we navigate from Genomics > Transcriptomics > Proteomics > Metabolomics. Sometimes the problem arises when trying to integrate information that is not easy to merge. Genomics provides a “full” overview of the system, while for example metabolomics can render very specific information. Some authors have used a combination of targeted amplicon sequencing and metabolomic profiling. This approach provides the taxonomical overview of the complex microbial community, while it also informs of the specific metabolic events taking place.

**Table 2:** Summary of studies involving use of one or more omics methodologies and the statistical data reduction commonly applied.

Ref.	Omics field	Study subject	Subject type	Experiment	Highlighted number of features	Data reduction
Imdahl et al. (2020)	Transcriptomics	*Salmonella enterica* serovar Typhimurium	Bacteria	Late stationary phase, anaerobic shock, NaCl shock	Pool: 101 (anaer.); 274 (NaCl) Single c.: 63 (anaer.); 131 (NaCl)	Unbiased clustering by PCA, DEGs (DESeq2)
Laguna-Teno et al. (2020)	Transcriptomics	*Saccharomyces cerevisiae*	Fungi/yeast	Toxicology exposure, two GOs vs. control	1,181 (GOC); 340 (GO)	PCA, DEGs, FDR, FC, functional annotation
Semmouri et al. (2020)	Transcriptomics	Zooplankton community	Environmental community	Seasonal community characterization	Over 3,000 annotated transcripts per sample	Functional interpretation
Bengtsson et al. (2016)	Proteomics	*Hypocrea jecorina*	Fungi	Secretome characterization	155 identified proteins	Hierarchical clustering
Bona et al. (2019)	Proteomics	*Vitis vinifera* cv. Pinot Noir rhizosphere	Environmental community	Proteome and taxonomy characterization	579 identified proteins	Functional interpretation
Knox et al. (2016)	Proteomics	*Aspergillus fumigatus*	Fungi	ISS strains vs. clinical isolates	N/S	One-way ANOVA, comparison of fold change (Log2)
Ram et al. (2005)	Genomics Proteomics	Natural acid mine drainage biofilm	Environmental community	Characterization of a naturally occurring biofilm	2,003 identified proteins	Functional interpretation
Zhang et al. (2014)	Proteomics	*Shewanella oneidensis*	Bacteria	Alginate entrapped cultures vs. biofilms	1,712 identified proteins	Student’s *t*-test
Khakimov et al. (2017)	Metabolomics	*Streptoccoccus thermophilus*	Bacteria	Several time points through fermentation process	64 identified peaks	PARAFAC2, PCA, ASCA
Favre et al. (2017)	Metabolomics	*Persicivirga* (Nonlabens) *mediterranea*; *Pseudoalteromonas lipolytica*; *Shewanella* sp.	Bacteria	Characterization of response to culture medium, growth phase, culture mode	155 ± 22 *m/z* features	PCA, PLS-DA
Lu et al. (2019)	Metabolomics	Uropathogenic *Escherichia coli*	Bacteria	Comparison between biofilm and planktonic stages	38 differential metabolites	Unsupervised PCA, PLS-DA, heatmap
Stipetic et al. (2016)	Metabolomics	*Staphylococcus aureus*	Bacteria	Comparison between biofilm and planktonic stages	530 significant metabolites	Bayes moderated *t*-tests, FDR, PCA
Afshari et al. (2020)	Genomics Metabolomics	Cheddar cheese microbial community	Environmental community	Comparison between industrial vs. traditional cheese, outer surface vs. core	46 metabolites (GC–MS) 8,000 features (LC–MS)	ANOSIM, PCA, MCIA (integration), Spearman analysis
Parrot et al. (2019)	Genomics Metabolomics Imaging	*Fucus vesiculosus* surface microbiome	Environmental community	Comparison of surface metabolome with whole tissue extract	50 metabolites (surface) 27 metabolites (extracts)	One-way PERMANOVA, PCoA, HAC

PCA, principal component analysis; DEGs, differentially expressed genes; Ref., reference; FC, fold change; ISS, international space station; N/S, not stated; N/A, not applicable; Single c., single cell; anaer., anaerobic; ASCA, ANOVA-simultaneous component analysis; PLS-DA, partial least-squares discriminate analysis; FDR, Benjamini and Hochberg false discovery rate; MCIA, multiple co-inertia analysis; HAC, hierarchical ascendant classification; PCoA, principal coordinates analysis; ASV, amplicon sequence variant; WWTP, wastewater treatment plant.

For long time mRNA levels recorded by transcriptomics have been used as a proxy for the protein levels. However, absolute levels of mRNA molecules and corresponding proteins are usually poorly correlated as the recorded changes (increase or decrease) in transcripts are buffered at the protein level through multiple levels of regulation (post-transcriptional, synthesis and degradation of proteins) (Greenbaum et al., 2003). The combination of multiple approaches can also focus the attention on regulatory events. As the use of multi-omics approaches gained momentum, statistical approaches have been deployed to reduce the dimensionality of the data and identify significant associations. Correlation, either Pearson or, most often, Spearman is commonly used for this task (Ni et al., 2020). Other approaches use multivariate analysis tools and the MixOmics R package (Rohart et al., 2017)^5^ provides an interface to these approaches. To gain insight in *Clostridioides difficile* nosocomial infection, Stewart et al. (Stewart et al., 2019) used MixOmics to integrate metagenomics and metatranscriptomics data in combination with the multivariate dimensionality-reduction tool, PLS-DA. Thanks to this unifying approach they characterized a transkingdom interaction between bacteria and fungi thus revealing antibiotic-independent mechanisms opposing the return to a healthy gut microbiome (Stewart et al., 2019). Paix et al. (2019) integrated metabolomics seasonal variation with metabarcoding. Data was integrated through a multi-block data integration analysis (DIABLO) (Singh et al., 2019), to establish a group of metabolites and OTUs that together could discriminate between months. In another integration study, Paix et al. (2020) used a distance-based redundancy analysis method to understand functional characteristics of a microbiome growing on a biotic surface (algae surface). The study associated microbial composition obtained through 16S rRNA sequencing and characterized using the UniFrac distance to normalize concentrations of relevant metabolites.

In addition to these statistical methods, mathematical models of metabolism can be used to describe metabolic phenotypes and provide a framework for integration of omics data, especially those that are difficult to integrate, such as genomic datasets. Constraint-based modeling is arguably the most widely adopted approach to represent cellular metabolism through Genome Scale Models (GEM) that represent the collection of metabolic reactions of an organism (Orth et al., 2010). GEMs are based on collecting genome encoded metabolic information in a stoichiometric matrix that can be used for quantitative and qualitative phenotype predictions. The models can be further adapted to specific conditions through the integration of transcriptional data [see (Sinha et al., 2021) and reference therein]. Such integration allows exploration of otherwise non-accessible measurements, such as the intracellular changes of *Burkholderia cenocepacia* along the different stages of biofilm development (Altay et al., 2021). Proteomics data provide an even closer link to the actual fluxes to obtain a GEM better adapted to a physiological state as in the case of the study of pH adaptation in the human pathogen *Enterococcus faecalis* (Großeholz et al., 2016).

## Conclusion

Where life exists, interactions are intrinsically set in an interwoven display of possibilities. We can study from the prey–predator relationships in the savanna, down to the realms of microbial species altering their molecular behavior in radical ways when encountering the boundaries of biotic or abiotic surfaces.

Omics technologies offer the opportunity to study surface-induced changes at an unprecedented resolution: a global scale. Preparation and analysis of the samples have greatly improved, and many methods have been developed to avoid contamination and preserve the biological information. Whether our interest lies in DNA, RNA, proteins or metabolites, obstacles are still present in recovering material from difficult environments and/or samples in sufficient quantity and quality. Miniaturization and automation of the sample preparation approaches will contribute greatly to alleviate these problems as well as the possibility of performing some type of omics analysis *in loco*, i.e., through portable sequencers. A still standing challenge is the development of a sample preparation method that could efficiently extract all types of biological material for the integrative analysis of all omics on the same specimen. Finally, we also look forward to the implementation of single cell omics technologies in the field with the confidence that adding such resolution will uncover key differences in the biological changes induced by direct and indirect surface interactions within a microorganismal community.

Multiomics approaches are well suited to reach a systems-level understanding of these biological processes and the underlying mechanisms behind them. The four types of studied molecules provide clues not only from a curiosity-driven scientific perspective, but also from an applied point of view. In the present review we have introduced the latest research trends in the field from the perspective of the four main omics technologies. Genomics can accurately answer questions about the identity of those cells colonizing the surface of interest and provide an overview of the potential metabolic and information lifestyle. At a deeper level, transcriptomics can assist in the evaluation of which genes from the genomic arsenal are put at play, while proteomics indicates accurately who is involved. DNA and proteins have a complementary key role in the maintenance of structures, as it has been explained in the case of the extracellular biofilm matrix. Metabolomics not only provides information about primary basic metabolism of cells, but also about the very important secondary metabolites. It has been shown how the metabolic fingerprint differs when cells are isolated and in a planktonic state, or when attached to surfaces and potentially interact with one another. The methodological approaches are, in most cases, directed toward the recovery of small biomass amounts.

Omics technologies largely depend on computational methodologies and bioinformatic pipelines to analyze the generated raw data and extract biological knowledge. Pipelines for data analysis often combine methods and algorithms specific for the chosen data type (such as mapping algorithms for RNA sequencing reads or tools for peak characterization for MS data analysis) with (statistical) methods that can be applied to a broad set of data types, such as PCA, PLS or enrichment analysis, as shown it Table 2. It should be noted that the cases we have reviewed show that standard bioinformatic pipelines are suitable to understand data generated from cells interacting with complex extracellular matrices, provided the sample acquisition process has generated material with sufficient quality. We believe that it can be advantageous to complement these pipelines with additional analysis to specifically capture the complexity of cell-surface interactions. We see great potential in methods to predict subcellular location that will allow extensive characterization of the microbial secretome and surfaceome. Finally, predictive algorithms to characterize protein–protein interactions can boost our understanding of the molecular mechanisms underlying cell–cell interactions.

Methods have been developed to model bacterial growth on surfaces, attachment and biofilm formation and to describe the physical properties of the system, see for instance (Horn and Lackner, 2014). To go one step further, approaches have been proposed to link the intracellular metabolism with the environmental changes due to the cell-surface interaction and associated gradients for organisms such as *E. coli* and *P. aeruginosa* (Biggs and Papin, 2013; Tack et al., 2017). Modeling frameworks, able to account for more complex systems and gradients, and with multiple species, are actively developed (Harcombe et al., 2014; Bauer et al., 2017; Angeles-Martinez and Hatzimanikatis, 2021; Dukovski et al., 2021). We believe omics datasets can be used to extensively test the predictive power of these methods and to further refine and increase their prediction accuracy. Accurate predictions of microbial behavior upon cell-surface interactions will provide a better understanding of the dynamics of colonization, will inform on the effect of environmental changes such as changes of nutrient availability, antibiotic addition, or introduction of additional microbial species, and will provide efficient means to prevent or exploit these phenomena.

## Author contributions

JG-P and CF: conceptualization, roles, writing — original draft, writing — review and editing, and visualization. TR, AL, and JT-R: conceptualization, roles, writing — original draft, and funding acquisition. RB: conceptualization and funding acquisition. MS-D: conceptualization, roles, writing — original draft, writing — review and editing, and funding acquisition. All authors contributed to the article and approved the submitted version.

## Funding

This project has received funding under the European Union’s Horizon 2020 research & Innovation programme under grant agreement no. 952379, SURFBIO project. JG-P is currently supported by Junta de Castilla y León-FEDER under grant N° BU058P20 (NANOCOMP).

## Conflict of interest

The authors declare that the research was conducted in the absence of any commercial or financial relationships that could be construed as a potential conflict of interest.

## Publisher’s note

All claims expressed in this article are solely those of the authors and do not necessarily represent those of their affiliated organizations, or those of the publisher, the editors and the reviewers. Any product that may be evaluated in this article, or claim that may be made by its manufacturer, is not guaranteed or endorsed by the publisher.

## Footnotes

^1^ https://metlin.scripps.edu/landing_page.php?pgcontent=mainPage

^2^ www.genome.jp/kegg/compound/

^3^ www.chemspider.com/

^4^ www.lipidmaps.org/

^5^ http://mixomics.org/

## References

[ref1] Afshari R. Pillidge C. J. Read E. Rochfort S. Dias D. A. Osborn A. M.. (2020). New insights into cheddar cheese microbiota-metabolome relationships revealed by integrative analysis of multi-omics data. Sci. Rep. 10:3164. doi: 10.1038/s41598-020-59617-9, PMID: 32081987 PMC7035325

[ref2] Agrawal S. Kumar S. Sehgal R. George S. Gupta R. Poddar S.. (2019). “El-MAVEN: a fast, robust, and user-friendly mass spectrometry data processing engine for Metabolomics” in High-Throughput Metabolomics: Methods and Protocols. ed. D’Alessandro A. (New York, NY: Springer), 301–321. 10.1007/978-1-4939-9236-2_19 31119671

[ref3] Alballa M. Butler G. (2020). Integrative approach for detecting membrane proteins. BMC Bioinformatics 21:575. doi: 10.1186/s12859-020-03891-x, PMID: 33349234 PMC7751106

[ref4] Alessi A. M. Bird S. M. Bennett J. P. Oates N. C. Li Y. Dowle A. A.. (2017). Revealing the insoluble metasecretome of lignocellulose-degrading microbial communities. Sci. Rep. 7:2356. doi: 10.1038/s41598-017-02506-5, PMID: 28539641 PMC5443780

[ref5] Altay O. Zhang C. Turkez H. Nielsen J. Uhlén M. Mardinoglu A. (2021). Revealing the metabolic alterations during biofilm development of Burkholderia cenocepacia based on genome-scale metabolic modeling. Meta 11:221. doi: 10.3390/metabo11040221, PMID: 33916474 PMC8067366

[ref6] Amarasinghe S. L. Su S. Dong X. Zappia L. Ritchie M. E. Gouil Q. (2020). Opportunities and challenges in long-read sequencing data analysis. Genome Biol. 21:30. doi: 10.1186/s13059-020-1935-5, PMID: 32033565 PMC7006217

[ref7] Angelakis E. Bachar D. Henrissat B. Armougom F. Audoly G. Lagier J. C.. (2016). Glycans affect DNA extraction and induce substantial differences in gut metagenomic studies. Sci. Rep. 6:26276. doi: 10.1038/srep26276, PMID: 27188959 PMC4870698

[ref8] Angeles-Martinez L. Hatzimanikatis V. (2021). The influence of the crowding assumptions in biofilm simulations. PLoS Comput. Biol. 17:e1009158. doi: 10.1371/journal.pcbi.1009158, PMID: 34292941 PMC8297847

[ref9] Arciola C. R. Campoccia D. Montanaro L. (2018). Implant infections: adhesion, biofilm formation and immune evasion. Nat. Rev. Microbiol. 16, 397–409. doi: 10.1038/s41579-018-0019-y, PMID: 29720707

[ref10] Azhikina T. L. Skvortsov T. A. Radaeva T. V. Mardanov A. V. Ravin N. V. Apt A. S.. (2010). A new technique for obtaining whole pathogen transcriptomes from infected host tissues. BioTechniques 48, 139–144. doi: 10.2144/000113350, PMID: 20359298 PMC2905682

[ref11] Bachmann R. T. Edyvean R. G. J. (2005). Biofouling: an historic and contemporary review of its causes, consequences and control in drinking water distribution systems. Biofilms 2, 197–227. doi: 10.1017/S1479050506001979

[ref12] Balayan A. Kapoor A. Chaudhary G. Raina A. (2015). Evaluation of techniques for human bone decalcification and amplification using sixteen STR markers. Egypt. J. Forensic Sci. 5, 30–35. doi: 10.1016/j.ejfs.2014.05.002

[ref13] Barbieri R. Mekni R. Levasseur A. Chabrière E. Signoli M. Tzortzis S.. (2017). Paleoproteomics of the dental pulp: the plague paradigm. PLoS One 12:e0180552. doi: 10.1371/journal.pone.0180552, PMID: 28746380 PMC5528255

[ref14] Bastida F. Hernández T. García C. (2014). Metaproteomics of soils from semiarid environment: functional and phylogenetic information obtained with different protein extraction methods. J. Proteome 101, 31–42. doi: 10.1016/j.jprot.2014.02.006, PMID: 24530626

[ref15] Bauer E. Zimmermann J. Baldini F. Thiele I. Kaleta C. (2017). BacArena: individual-based metabolic modeling of heterogeneous microbes in complex communities. PLoS Comput. Biol. 13:e1005544. doi: 10.1371/journal.pcbi.1005544, PMID: 28531184 PMC5460873

[ref16] Beale D. J. Karpe A. V. Jadhav S. Muster T. H. Palombo E. A. (2016). Omics-based approaches and their use in the assessment of microbial-influenced corrosion of metals. Corros. Rev. 34, 1–15. doi: 10.1515/corrrev-2015-0046

[ref17] Bengtsson O. Arntzen M. Ø. Mathiesen G. Skaugen M. Eijsink V. G. H. (2016). A novel proteomics sample preparation method for secretome analysis of *Hypocrea jecorina* growing on insoluble substrates. J. Proteome 131, 104–112. doi: 10.1016/j.jprot.2015.10.017, PMID: 26477388

[ref18] Berne C. Ellison C. K. Ducret A. Brun Y. V. (2018). Bacterial adhesion at the single-cell level. Nat. Rev. Microbiol. 16, 616–627. doi: 10.1038/s41579-018-0057-5, PMID: 30008468

[ref19] Bharti R. Grimm D. G. (2021). Current challenges and best-practice protocols for microbiome analysis. Brief. Bioinform. 22, 178–193. doi: 10.1093/bib/bbz155, PMID: 31848574 PMC7820839

[ref20] Biggs M. B. Papin J. A. (2013). Novel multiscale modeling tool applied to *Pseudomonas aeruginosa* biofilm formation. PLoS One 8:e78011. doi: 10.1371/journal.pone.0078011, PMID: 24147108 PMC3798466

[ref21] Blachowicz A. Chiang A. J. Romsdahl J. Kalkum M. Wang C. C. C. Venkateswaran K. (2019). Proteomic characterization of *Aspergillus fumigatus* isolated from air and surfaces of the international space station. Fungal Genet. Biol. 124, 39–46. doi: 10.1016/j.fgb.2019.01.001, PMID: 30611835 PMC9116463

[ref22] Blakeley-Ruiz J. A. Kleiner M. (2022). Considerations for constructing a protein sequence database for metaproteomics. Comput. Struct. Biotechnol. J. 20, 937–952. doi: 10.1016/j.csbj.2022.01.018, PMID: 35242286 PMC8861567

[ref23] Blankenburg S. Hentschker C. Nagel A. Hildebrandt P. Michalik S. Dittmar D.. (2019). Improving proteome coverage for small sample amounts: an advanced method for proteomics approaches with low bacterial cell numbers. Proteomics 19:1900192. doi: 10.1002/pmic.201900192, PMID: 31532911

[ref24] Bona E. Massa N. Novello G. Boatti L. Cesaro P. Todeschini V.. (2019). Metaproteomic characterization of the *Vitis vinifera* rhizosphere. FEMS Microbiol. Ecol. 95:fiy204. doi: 10.1093/femsec/fiy204, PMID: 30307579

[ref25] Bonn F. Maaß S. van Dijl J. M. (2018). Enrichment of cell surface-associated proteins in gram-positive bacteria by Biotinylation or trypsin shaving for mass spectrometry analysis. Methods Mol. Biol. 1841, 35–43. doi: 10.1007/978-1-4939-8695-8_4, PMID: 30259478

[ref26] Booncharoen P. Khacha-ananda S. Kanchai C. Ruengdit S. (2021). Factors influencing DNA extraction from human skeletal remains: bone characteristic and total demineralization process. Egypt. J. Forensic Sci. 11:2. doi: 10.1186/s41935-021-00216-8

[ref27] Boyce J. M. (2016). Modern technologies for improving cleaning and disinfection of environmental surfaces in hospitals. Antimicrob. Resist. Infect. Control 5:10. doi: 10.1186/s13756-016-0111-x, PMID: 27069623 PMC4827199

[ref28] Brauer J. I. Celikkol-Aydin S. Sunner J. A. Gaylarde C. C. Beech I. B. (2017). Metabolomic imaging of a quaternary ammonium salt within a marine bacterial biofilm on carbon steel. Int. Biodeterior. Biodegradation 125, 33–36. doi: 10.1016/j.ibiod.2017.08.007

[ref29] Buckley M. (2019). “Paleoproteomics: an introduction to the analysis of ancient proteins by soft ionisation mass spectrometry” in Paleogenomics: Genome-Scale Analysis of Ancient DNA. eds. Lindqvist C. Rajora O. P. (Cham: Springer International Publishing), 31–52.

[ref30] Bürgers R. Morsczeck C. Felthaus O. Gosau M. Beck H. C. Reichert T. E. (2018). Induced surface proteins of staphylococcus [corrected] epidermidis adhering to titanium implant substrata. Clin. Oral Investig. 22, 2663–2668. doi: 10.1007/s00784-018-2508-9 29948278

[ref31] Cassidy S. S. Sanders D. J. Wade J. Parkin I. P. Carmalt C. J. Smith A. M.. (2020). Antimicrobial surfaces: a need for stewardship? PLoS Pathog. 16:e1008880. doi: 10.1371/journal.ppat.1008880, PMID: 33057433 PMC7561179

[ref32] Cennamo P. Montuori N. Trojsi G. Fatigati G. Moretti A. (2016). Biofilms in churches built in grottoes. Sci. Total Environ. 543, 727–738. doi: 10.1016/j.scitotenv.2015.11.048, PMID: 26618300

[ref33] Chapelle E. Alunni B. Malfatti P. Solier L. Pédron J. Kraepiel Y.. (2015). A straightforward and reliable method for bacterial in planta transcriptomics: application to the Dickeya dadantii/Arabidopsis thaliana pathosystem. Plant J. 82, 352–362. doi: 10.1111/tpj.12812, PMID: 25740271

[ref34] Chavez-Dozal A. Gorman C. Nishiguchi M. K. (2015). Proteomic and metabolomic profiles demonstrate variation among free-living and symbiotic vibrio fischeri biofilms. BMC Microbiol. 15:226. doi: 10.1186/s12866-015-0560-z, PMID: 26494154 PMC4619220

[ref35] Chen Z. Chen L. Zhang W. (2017). Tools for genomic and Transcriptomic analysis of microbes at single-cell level. Front. Microbiol. 8:1831. doi: 10.3389/fmicb.2017.01831, PMID: 28979258 PMC5611438

[ref36] Chen Y. Guenther J. M. Gin J. W. Chan L. J. G. Costello Z. Ogorzalek T. L.. (2019). Automated ‘cells-to-peptides’ sample preparation workflow for high-throughput, quantitative proteomic assays of microbes. J. Proteome Res. 18, 3752–3761. doi: 10.1021/acs.jproteome.9b00455, PMID: 31436101

[ref37] Cheng K. Brunius C. Fristedt R. Landberg R. (2020). An LC-QToF MS based method for untargeted metabolomics of human fecal samples. Metabolomics 16:46. doi: 10.1007/s11306-020-01669-z, PMID: 32246267 PMC7125068

[ref38] Chourey K. Hettich R. L. (2018). Utilization of a detergent-based method for direct microbial cellular Lysis/proteome extraction from soil samples for Metaproteomics studies. Methods Mol. Biol. 1841, 293–302. doi: 10.1007/978-1-4939-8695-8_20, PMID: 30259494

[ref39] Chung M. Bruno V. M. Rasko D. A. Cuomo C. A. Muñoz J. F. Livny J.. (2021). Best practices on the differential expression analysis of multi-species RNA-seq. Genome Biol. 22:121. doi: 10.1186/s13059-021-02337-8, PMID: 33926528 PMC8082843

[ref40] Cirri E. Grosser K. Pohnert G. (2016). A solid phase extraction based non-disruptive sampling technique to investigate the surface chemistry of macroalgae. Biofouling 32, 145–153. doi: 10.1080/08927014.2015.1130823, PMID: 26795737

[ref41] Ciuffreda L. Rodríguez-Pérez H. Flores C. (2021). Nanopore sequencing and its application to the study of microbial communities. Comput. Struct. Biotechnol. J. 19, 1497–1511. doi: 10.1016/j.csbj.2021.02.020, PMID: 33815688 PMC7985215

[ref42] Clark K. D. Nacham O. Yu H. Li T. Yamsek M. M. Ronning D. R.. (2015). Extraction of DNA by magnetic ionic liquids: tunable solvents for rapid and selective DNA analysis. Anal. Chem. 87, 1552–1559. doi: 10.1021/ac504260t, PMID: 25582771

[ref43] Clasquin M. F. Melamud E. Rabinowitz J. D. (2012). LC-MS data processing with MAVEN: a metabolomic analysis and visualization engine. Curr. Protoc. Bioinformatics Chapter 14:Unit14.11. doi: 10.1002/0471250953.bi1411s37, PMID: 22389014 PMC4055029

[ref44] Cobrado L. Silva-Dias A. Azevedo M. M. Rodrigues A. G. (2017). High-touch surfaces: microbial neighbours at hand. Eur. J. Clin. Microbiol. Infect. Dis. 36, 2053–2062. doi: 10.1007/s10096-017-3042-4, PMID: 28647859 PMC7087772

[ref45] Coetser S. E. Cloete T. E. (2005). Biofouling and biocorrosion in industrial water systems. Crit. Rev. Microbiol. 31, 213–232. doi: 10.1080/10408410500304074, PMID: 16417202

[ref46] Corchete L. A. Rojas E. A. Alonso-López D. De Las Rivas J. Gutiérrez N. C. Burguillo F. J. (2020). Systematic comparison and assessment of RNA-seq procedures for gene expression quantitative analysis. Sci. Rep. 10:19737. doi: 10.1038/s41598-020-76881-x, PMID: 33184454 PMC7665074

[ref47] da Costa Rosa T. de Almeida Neves A. Azcarate-Peril M. A. Divaris K. Wu D. Cho H.. (2021). The bacterial microbiome and metabolome in caries progression and arrest. J. Oral Microbiol. 13:1886748. doi: 10.1080/20002297.2021.1886748, PMID: 34188775 PMC8211139

[ref48] de Vries H. J. Kleibusch E. Hermes G. D. A. van den Brink P. Plugge C. M. (2021). Biofouling control: the impact of biofilm dispersal and membrane flushing. Water Res. 198:117163. doi: 10.1016/j.watres.2021.117163, PMID: 33951583

[ref49] Deev D. Rybkin I. Rijavec T. Lapanje A. (2021). When beneficial biofilm on materials is needed: electrostatic attachment of living bacterial cells induces biofilm formation. Front. Mater. 8:624631. doi: 10.3389/fmats.2021.624631

[ref50] Demain A. L. (1980). Microbial production of primary metabolites. Naturwissenschaften 67, 582–587. doi: 10.1007/BF00396537 7231563

[ref51] Dengler V. Foulston L. DeFrancesco A. S. Losick R. (2015). An electrostatic net model for the role of extracellular DNA in biofilm formation by *Staphylococcus aureus*. J. Bacteriol. 197, 3779–3787. doi: 10.1128/JB.00726-15, PMID: 26416831 PMC4652055

[ref52] Ding X. Lan W. Li Y. Yan A. Katayama Y. Koba K.. (2021). An internal recycling mechanism between ammonia/ammonium and nitrate driven by ammonia-oxidizing archaea and bacteria (AOA, AOB, and Comammox) and DNRA on Angkor sandstone monuments. Int. Biodeterior. Biodegradation 165:105328. doi: 10.1016/j.ibiod.2021.105328

[ref53] Domingo-Almenara X. Brezmes J. Vinaixa M. Samino S. Ramirez N. Ramon-Krauel M.. (2016). eRah: a computational tool integrating spectral Deconvolution and alignment with quantification and identification of metabolites in GC/MS-based Metabolomics. Anal. Chem. 88, 9821–9829. doi: 10.1021/acs.analchem.6b02927, PMID: 27584001

[ref54] Dominguez Del Angel V. Hjerde E. Sterck L. Capella-Gutierrez S. Notredame C. Vinnere Pettersson O.. (2018). Ten steps to get started in genome assembly and annotation. F1000Res 7, ELIXIR–148. doi: 10.12688/f1000research.13598.1, PMID: 29568489 PMC5850084

[ref55] Dukovski I. Bajić D. Chacón J. M. Quintin M. Vila J. C. C. Sulheim S.. (2021). A metabolic modeling platform for the computation of microbial ecosystems in time and space (COMETS). Nat. Protoc. 16, 5030–5082. doi: 10.1038/s41596-021-00593-3, PMID: 34635859 PMC10824140

[ref56] Dupre J. M. Johnson W. L. Ulanov A. V. Li Z. Wilkinson B. J. Gustafson J. E. (2019). Transcriptional profiling and metabolomic analysis of *Staphylococcus aureus* grown on autoclaved chicken breast. Food Microbiol. 82, 46–52. doi: 10.1016/j.fm.2019.01.004 31027806

[ref57] Eisenhofer R. Minich J. J. Marotz C. Cooper A. Knight R. Weyrich L. S. (2019). Contamination in low microbial biomass microbiome studies: issues and recommendations. Trends Microbiol. 27, 105–117. doi: 10.1016/j.tim.2018.11.003, PMID: 30497919

[ref58] Ejigu G. F. Jung J. (2020). Review on the computational genome annotation of sequences obtained by next-generation sequencing. Biology (Basel) 9:295. doi: 10.3390/biology9090295, PMID: 32962098 PMC7565776

[ref59] Emaus M. N. Varona M. Eitzmann D. R. Hsieh S.-A. Zeger V. R. Anderson J. L. (2020). Nucleic acid extraction: fundamentals of sample preparation methodologies, current advancements, and future endeavors. TrAC Trends Anal. Chem. 130:115985. doi: 10.1016/j.trac.2020.115985

[ref60] Emwas A.-H. Roy R. McKay R. Tenori L. Saccenti E. Gowda G. A. N.. (2019). NMR spectroscopy for metabolomics research. Meta 9:123. doi: 10.3390/metabo9070123, PMID: 31252628 PMC6680826

[ref61] Erde J. Loo R. R. O. Loo J. A. (2014). Enhanced FASP (eFASP) to increase proteome coverage and sample recovery for quantitative proteomic experiments. J. Proteome Res. 13, 1885–1895. doi: 10.1021/pr4010019, PMID: 24552128 PMC3993969

[ref62] Farrer A. G. Wright S. L. Skelly E. Eisenhofer R. Dobney K. Weyrich L. S. (2021). Effectiveness of decontamination protocols when analyzing ancient DNA preserved in dental calculus. Sci. Rep. 11:7456. doi: 10.1038/s41598-021-86100-w, PMID: 33811235 PMC8018977

[ref63] Favre L. Ortalo-Magné A. Greff S. Pérez T. Thomas O. P. Martin J. C.. (2017). Discrimination of four marine biofilm-forming bacteria by LC–MS metabolomics and influence of culture parameters. J. Proteome Res. 16, 1962–1975. doi: 10.1021/acs.jproteome.6b01027, PMID: 28362105

[ref64] Favre L. Ortalo-Magné A. Pichereaux C. Gargaros A. Burlet-Schiltz O. Cotelle V.. (2018). Metabolome and proteome changes between biofilm and planktonic phenotypes of the marine bacterium *Pseudoalteromonas lipolytica* TC8. Biofouling 34, 132–148. doi: 10.1080/08927014.2017.1413551, PMID: 29319346

[ref65] Fiehn O. Kopka J. Dörmann P. Altmann T. Trethewey R. N. Willmitzer L. (2000). Metabolite profiling for plant functional genomics. Nat. Biotechnol. 18, 1157–1161. doi: 10.1038/81137 11062433

[ref66] Flemming H.-C. (2020). Biofouling and me: my Stockholm syndrome with biofilms. Water Res. 173:115576. doi: 10.1016/j.watres.2020.115576, PMID: 32044598

[ref67] Flemming H. C. Wingender J. Szewzyk U. Steinberg P. Rice S. A. Kjelleberg S. (2016). Biofilms: an emergent form of bacterial life. Nat. Rev. Microbiol. 14, 563–575. doi: 10.1038/NRMICRO.2016.94, PMID: 27510863

[ref68] Fordyce S. L. Ávila-Arcos M. C. Rasmussen M. Cappellini E. Romero-Navarro J. A. Wales N.. (2013). Deep sequencing of RNA from ancient maize kernels. PLoS One 8:e50961. doi: 10.1371/journal.pone.0050961, PMID: 23326310 PMC3543400

[ref69] França A. Melo L. D. Cerca N. (2011). Comparison of RNA extraction methods from biofilm samples of Staphylococcus epidermidis. BMC. Res. Notes 4:572. doi: 10.1186/1756-0500-4-572, PMID: 22208502 PMC3260333

[ref70] Gardinassi L. G. Xia J. Safo S. E. Li S. (2017). Bioinformatics tools for the interpretation of Metabolomics data. Curr Pharmacol Rep 3, 374–383. doi: 10.1007/s40495-017-0107-0

[ref71] Genova C. Grottoli A. Zoppis E. Cencetti C. Matricardi P. Favero G. (2020). An integrated approach to the recovery of travertine biodegradation by combining phyto-cleaning with genomic characterization. Microchem. J. 156:104918. doi: 10.1016/j.microc.2020.104918

[ref72] Giacomoni F. le Corguille G. Monsoor M. Landi M. Pericard P. Petera M.. (2015). Workflow4Metabolomics: a collaborative research infrastructure for computational metabolomics. Bioinformatics 31, 1493–1495. doi: 10.1093/bioinformatics/btu813, PMID: 25527831 PMC4410648

[ref73] Giani A. M. Gallo G. R. Gianfranceschi L. Formenti G. (2019). Long walk to genomics: history and current approaches to genome sequencing and assembly. Comput. Struct. Biotechnol. J. 18, 9–19. doi: 10.1016/j.csbj.2019.11.002, PMID: 31890139 PMC6926122

[ref74] Gika H. Theodoridis G. (2011). Sample preparation prior to the LC–MS-based metabolomics/metabonomics of blood-derived samples. Bioanalysis 3, 1647–1661. doi: 10.4155/bio.11.122, PMID: 21756097

[ref75] Gilbert P. Das J. Foley I. (1997). Biofilm susceptibility to antimicrobials. Adv. Dent. Res. 11, 160–167. doi: 10.1177/08959374970110010701 9524452

[ref76] Gil-Bona A. Bidlack F. B. (2020). Tooth enamel and its dynamic protein matrix. Int. J. Mol. Sci. 21:4458. doi: 10.3390/ijms21124458, PMID: 32585904 PMC7352428

[ref77] Gloag E. S. Fabbri S. Wozniak D. J. Stoodley P. (2020). Biofilm mechanics: implications in infection and survival. Biofilms 2:100017. doi: 10.1016/j.bioflm.2019.100017, PMID: 33447803 PMC7798440

[ref78] Gong Z.-G. Hu J. Wu X. Xu Y.-J. (2017). The recent developments in sample preparation for mass spectrometry-based Metabolomics. Crit. Rev. Anal. Chem. 47, 325–331. doi: 10.1080/10408347.2017.1289836, PMID: 28631936

[ref79] González J. M. Sáiz-Jiménez C. (2005). Application of molecular nucleic acid-based techniques for the study of microbial communities in monuments and artworks. Int. Microbiol. 8, 189–194. PMID: 16200497

[ref80] Gostine A. Gostine D. Short J. Rustagi A. Cadnum J. Donskey C.. (2020). Evaluating the utility of UV lamps to mitigate the spread of pathogens in the ICU. Appl. Sci. 10:18. doi: 10.3390/app10186326

[ref81] Graf A. C. Striesow J. Pané-Farré J. Sura T. Wurster M. Lalk M.. (2021). An innovative protocol for Metaproteomic analyses of microbial pathogens in cystic fibrosis sputum. Front. Cell. Infect. Microbiol. 11:809. doi: 10.3389/fcimb.2021.724569, PMID: 34513734 PMC8432295

[ref82] Greco E. el-Aguizy O. Ali M. F. Foti S. Cunsolo V. Saletti R.. (2018). Proteomic analyses on an ancient Egyptian cheese and biomolecular evidence of brucellosis. Anal. Chem. 90, 9673–9676. doi: 10.1021/acs.analchem.8b02535, PMID: 30044608

[ref83] Greenbaum D. Colangelo C. Williams K. Gerstein M. (2003). Comparing protein abundance and mRNA expression levels on a genomic scale. Genome Biol. 4:117. doi: 10.1186/gb-2003-4-9-117, PMID: 12952525 PMC193646

[ref84] Großeholz R. Koh C. C. Veith N. Fiedler T. Strauss M. Olivier B.. (2016). Integrating highly quantitative proteomics and genome-scale metabolic modeling to study pH adaptation in the human pathogen enterococcus faecalis. NPJ Syst Biol Appl 2:16017. doi: 10.1038/npjsba.2016.17, PMID: 28725473 PMC5516852

[ref85] Guilhen C. Charbonnel N. Parisot N. Gueguen N. Iltis A. Forestier C.. (2016). Transcriptional profiling of Klebsiella pneumoniae defines signatures for planktonic, sessile and biofilm-dispersed cells. BMC Genomics 17:237. doi: 10.1186/s12864-016-2557-x, PMID: 26979871 PMC4791964

[ref86] Guo X. S. Ke W. C. Ding W. R. Ding L. M. Xu D. M. Wang W. W.. (2018). Profiling of metabolome and bacterial community dynamics in ensiled Medicago sativa inoculated without or with lactobacillus plantarum or lactobacillus buchneri. Sci. Rep. 8:357. doi: 10.1038/s41598-017-18348-0, PMID: 29321642 PMC5762819

[ref87] Guo R. Luo X. Liu J. Lu H. (2021). Mass spectrometry based targeted metabolomics precisely characterized new functional metabolites that regulate biofilm formation in *Escherichia coli*. Anal. Chim. Acta 1145, 26–36. doi: 10.1016/j.aca.2020.12.021, PMID: 33453877

[ref88] Guy P. L. (2013). Ancient RNA? RT-PCR of 50-year-old RNA identifies peach latent mosaic viroid. Arch. Virol. 158, 691–694. doi: 10.1007/s00705-012-1527-0, PMID: 23138153

[ref89] Guy P. L. (2014). Prospects for analyzing ancient RNA in preserved materials. WIREs RNA 5, 87–94. doi: 10.1002/wrna.1199, PMID: 24343860

[ref90] Gwak H.-J. Lee S. J. Rho M. (2021). Application of computational approaches to analyze metagenomic data. J. Microbiol. 59, 233–241. doi: 10.1007/s12275-021-0632-8, PMID: 33565054

[ref91] Harcombe W. R. Riehl W. J. Dukovski I. Granger B. R. Betts A. Lang A. H.. (2014). Metabolic resource allocation in individual microbes determines ecosystem interactions and spatial dynamics. Cell Rep. 7, 1104–1115. doi: 10.1016/j.celrep.2014.03.070, PMID: 24794435 PMC4097880

[ref92] Harding J. L. Reynolds M. M. (2014). Combating medical device fouling. Trends Biotechnol. 32, 140–146. doi: 10.1016/j.tibtech.2013.12.004, PMID: 24438709

[ref93] Harrison P. W. Ahamed A. Aslam R. Alako B. T. F. Burgin J. Buso N.. (2021). The European nucleotide archive in 2020. Nucleic Acids Res. 49, D82–D85. doi: 10.1093/nar/gkaa1028, PMID: 33175160 PMC7778925

[ref94] Horai H. Arita M. Kanaya S. Nihei Y. Ikeda T. Suwa K.. (2010). MassBank: a public repository for sharing mass spectral data for life sciences. J. Mass Spectrom. 45, 703–714. doi: 10.1002/jms.1777, PMID: 20623627

[ref95] Horn H. Lackner S. (2014). Modeling of biofilm systems: a review. Adv. Biochem. Eng. Biotechnol. 146, 53–76. doi: 10.1007/10_2014_275, PMID: 25163572

[ref96] Hu T. Chitnis N. Monos D. Dinh A. (2021). Next-generation sequencing technologies: an overview. Hum. Immunol. 82, 801–811. doi: 10.1016/j.humimm.2021.02.012 33745759

[ref97] Hug L. A. Co R. (2018). It takes a village: microbial communities thrive through interactions and metabolic handoffs. mSystems 3:e00152-17. doi: 10.1128/mSystems.00152-17, PMID: 29556533 PMC5850073

[ref98] Hurley W. L. Finkelstein E. Holst B. D. (1985). Identification of surface proteins on bovine leukocytes by a biotin-avidin protein blotting technique. J. Immunol. Methods 85, 195–202. doi: 10.1016/0022-1759(85)90287-x, PMID: 4078309

[ref99] Ibáñez de Aldecoa A. L. Zafra O. González-Pastor J. E. (2017). Mechanisms and regulation of extracellular DNA release and its biological roles in microbial communities. Front. Microbiol. 8:1390. doi: 10.3389/fmicb.2017.01390, PMID: 28798731 PMC5527159

[ref100] Imai K. Nakai K. (2020). Tools for the recognition of sorting signals and the prediction of subcellular localization of proteins from their amino acid sequences. Front. Genet. 11:607812. doi: 10.3389/fgene.2020.607812, PMID: 33324450 PMC7723863

[ref101] Imdahl F. Vafadarnejad E. Homberger C. Saliba A.-E. Vogel J. (2020). Single-cell RNA-sequencing reports growth-condition-specific global transcriptomes of individual bacteria. Nat. Microbiol. 5, 1202–1206. doi: 10.1038/s41564-020-0774-1, PMID: 32807892

[ref102] Imperi F. Caneva G. Cancellieri L. Ricci M. A. Sodo A. Visca P. (2007). The bacterial aetiology of rosy discoloration of ancient wall paintings. Environ Microbiol 9, 2894–2902. doi: 10.1111/j.1462-2920.2007.01393.x 17922771

[ref103] Iwasaki Y. Sawada T. Hatayama K. Ohyagi A. Tsukuda Y. Namekawa K.. (2012). Separation technique for the determination of highly polar metabolites in biological samples. Meta 2, 496–515. doi: 10.3390/metabo2030496, PMID: 24957644 PMC3901216

[ref104] Jacyna J. Kordalewska M. Markuszewski M. J. (2019). Design of Experiments in metabolomics-related studies: an overview. J. Pharm. Biomed. Anal. 164, 598–606. doi: 10.1016/j.jpba.2018.11.027, PMID: 30469109

[ref105] Jain A. Singh H. B. Das S. (2021). Deciphering plant-microbe crosstalk through proteomics studies. Microbiol. Res. 242:126590. doi: 10.1016/j.micres.2020.126590, PMID: 33022544

[ref106] Jakubovics N. S. Shields R. C. Rajarajan N. Burgess J. G. (2013). Life after death: the critical role of extracellular DNA in microbial biofilms. Lett. Appl. Microbiol. 57, 467–475. doi: 10.1111/lam.12134, PMID: 23848166

[ref107] Jiang W. Qiu Y. Ni Y. Su M. Jia W. Du X. (2010). An automated data analysis pipeline for GC−TOF−MS Metabonomics studies. J. Proteome Res. 9, 5974–5981. doi: 10.1021/pr1007703, PMID: 20825247

[ref108] Jumper J. Evans R. Pritzel A. Green T. Figurnov M. Ronneberger O.. (2021). Highly accurate protein structure prediction with AlphaFold. Nature 596, 583–589. doi: 10.1038/s41586-021-03819-2, PMID: 34265844 PMC8371605

[ref109] Kamminga T. Benis N. Martins dos Santos V. Bijlsma J. J. E. Schaap P. J. (2020). Combined Transcriptome sequencing of mycoplasma hyopneumoniae and infected pig lung tissue reveals up-regulation of bacterial F1-like ATPase and Down-regulation of the P102 cilium Adhesin in vivo. Front. Microbiol. 11:1679. doi: 10.3389/fmicb.2020.01679, PMID: 32765473 PMC7379848

[ref110] Kanani H. Chrysanthopoulos P. K. Klapa M. I. (2008). Standardizing GC–MS metabolomics. J. Chromatogr. B 871, 191–201. doi: 10.1016/j.jchromb.2008.04.049, PMID: 18538643

[ref111] Kavanaugh J. S. Flack C. E. Lister J. Ricker E. B. Ibberson C. B. Jenul C.. (2019). Identification of extracellular DNA-binding proteins in the biofilm matrix. MBio 10:e01137-19. doi: 10.1128/mBio.01137-19, PMID: 31239382 PMC6593408

[ref112] Kayani M. U. R. Huang W. Feng R. Chen L. (2021). Genome-resolved metagenomics using environmental and clinical samples. Brief. Bioinform. 22:bbab030. doi: 10.1093/bib/bbab030, PMID: 33758906 PMC8425419

[ref113] Keiblinger K. M. Riedel K. (2018). “Sample preparation for Metaproteome analyses of soil and leaf litter” in Microbial proteomics: Methods and Protocols. ed. Becher D., vol. 1841 (New York, NY: Springer), 303–318. 10.1007/978-1-4939-8695-8_21 30259495

[ref114] Keller C. Wei P. Wancewicz B. Cross T.-W. L. Rey F. E. Li L. (2021). Extraction optimization for combined Metabolomics, Peptidomics, and proteomics analysis of gut microbiota samples. J. Mass Spectrom. 56:e4625. doi: 10.1002/jms.4625, PMID: 32885503 PMC7855350

[ref115] Kessner D. Chambers M. Burke R. Agus D. Mallick P. (2008). ProteoWizard: open source software for rapid proteomics tools development. Bioinformatics 24, 2534–2536. doi: 10.1093/bioinformatics/btn323, PMID: 18606607 PMC2732273

[ref116] Khakimov B. Christiansen L. D. Heins A. L. Sørensen K. M. Schöller C. Clausen A.. (2017). Untargeted GC-MS Metabolomics reveals changes in the metabolite dynamics of industrial scale batch fermentations of *Streptoccoccus thermophilus* broth. Biotechnol. J. 12:1700400. doi: 10.1002/biot.201700400, PMID: 29034577

[ref117] Kim H.-J. Park J. S. Lee T. K. Kang D. Kang J. H. Shin K.. (2021). Dynamics of marine bacterial biofouling communities after initial Alteromonas genovensis biofilm attachment to anti-fouling paint substrates. Mar. Pollut. Bull. 172:112895. doi: 10.1016/j.marpolbul.2021.112895, PMID: 34455348

[ref118] Kim S. Thiessen P. A. Bolton E. E. Chen J. Fu G. Gindulyte A.. (2016). PubChem substance and compound databases. Nucleic Acids Res. 44, D1202–D1213. doi: 10.1093/nar/gkv951, PMID: 26400175 PMC4702940

[ref119] Kimkes T. E. P. Heinemann M. (2020). How bacteria recognise and respond to surface contact. FEMS Microbiol. Rev. 44, 106–122. doi: 10.1093/femsre/fuz029, PMID: 31769807 PMC7053574

[ref120] Kind T. Liu K.-H. Lee D. Y. DeFelice B. Meissen J. K. Fiehn O. (2013). LipidBlast in silico tandem mass spectrometry database for lipid identification. Nat. Methods 10, 755–758. doi: 10.1038/nmeth.2551, PMID: 23817071 PMC3731409

[ref121] Knox B. P. Blachowicz A. Palmer J. M. Romsdahl J. Huttenlocher A. Wang C. C. C.. (2016). Characterization of *Aspergillus fumigatus* isolates from air and surfaces of the international Space Station. mSphere 1:00227-16. doi: 10.1128/mSphere.00227-16, PMID: 27830189 PMC5082629

[ref122] Kok M. G. M. Nix C. Nys G. Fillet M. (2019). Targeted metabolomics of whole blood using volumetric absorptive microsampling. Talanta 197, 49–58. doi: 10.1016/j.talanta.2019.01.014, PMID: 30771966

[ref123] Kopka J. Schauer N. Krueger S. Birkemeyer C. Usadel B. Bergmuller E.. (2005). GMD@CSB.DB: the Golm Metabolome database. Bioinformatics 21, 1635–1638. doi: 10.1093/bioinformatics/bti236, PMID: 15613389

[ref124] Krafft A. E. Duncan B. W. Bijwaard K. E. Taubenberger J. K. Lichy J. H. (1997). Optimization of the isolation and amplification of RNA from formalin-fixed, paraffin-embedded tissue: the armed forces Institute of Pathology Experience and Literature Review. Mol. Diagn. 2, 217–230. doi: 10.1054/MODI00200217, PMID: 10462613

[ref125] Kuffel A. Gray A. Daeid N. N. (2021). Impact of metal ions on PCR inhibition and RT-PCR efficiency. Int. J. Legal Med. 135, 63–72. doi: 10.1007/s00414-020-02363-4, PMID: 32621147 PMC7782418

[ref126] Kuhl C. Tautenhahn R. Böttcher C. Larson T. R. Neumann S. (2012). CAMERA: an integrated strategy for compound spectra extraction and annotation of liquid chromatography/mass spectrometry data sets. Anal. Chem. 84, 283–289. doi: 10.1021/ac202450g, PMID: 22111785 PMC3658281

[ref127] Laguna-Teno F. Suarez-Diez M. Tamayo-Ramos J. A. (2020). Commonalities and differences in the transcriptional response of the model fungus *Saccharomyces cerevisiae* to different commercial Graphene oxide materials. Front. Microbiol. 11:1943. doi: 10.3389/fmicb.2020.01943, PMID: 32849484 PMC7431627

[ref128] Lauder A. P. Roche A. M. Sherrill-Mix S. Bailey A. Laughlin A. L. Bittinger K.. (2016). Comparison of placenta samples with contamination controls does not provide evidence for a distinct placenta microbiota. Microbiome 4:29. doi: 10.1186/s40168-016-0172-3, PMID: 27338728 PMC4917942

[ref129] Leadbeater D. R. Oates N. C. Bennett J. P. Li Y. Dowle A. A. Taylor J. D.. (2021). Mechanistic strategies of microbial communities regulating lignocellulose deconstruction in a UK salt marsh. Microbiome 9:48. doi: 10.1186/s40168-020-00964-0, PMID: 33597033 PMC7890819

[ref130] Leventhal G. E. Boix C. Kuechler U. Enke T. N. Sliwerska E. Holliger C.. (2018). Strain-level diversity drives alternative community types in millimetre-scale granular biofilms. Nat. Microbiol. 3, 1295–1303. doi: 10.1038/s41564-018-0242-3, PMID: 30250246

[ref131] Li M. Wen J. (2021). Recent progress in the application of omics technologies in the study of bio-mining microorganisms from extreme environments. Microb. Cell Factories 20:178. doi: 10.1186/s12934-021-01671-7, PMID: 34496835 PMC8425152

[ref132] Li Y. Xiao P. Wang Y. Hao Y. (2020). Mechanisms and control measures of mature biofilm resistance to antimicrobial agents in the clinical context. ACS Omega 5, 22684–22690. doi: 10.1021/acsomega.0c02294, PMID: 32954115 PMC7495453

[ref133] Li Z. Yao Q. Guo X. Crits-Christoph A. Mayes M. A. IV W. J. H.. (2019). Genome-resolved proteomic stable isotope probing of soil microbial communities using 13CO2 and 13C-methanol. Front. Microbiol. 10:2706. doi: 10.3389/fmicb.2019.02706, PMID: 31866955 PMC6908837

[ref134] Liang S. Jiang W. Song Y. Zhou S.-F. (2020). Improvement and Metabolomics-based analysis of d-lactic acid production from agro-industrial wastes by lactobacillus delbrueckii submitted to adaptive laboratory evolution. J. Agric. Food Chem. 68, 7660–7669. doi: 10.1021/acs.jafc.0c00259, PMID: 32603099

[ref135] Liao Z.-X. Ni Z. Wei X. L. Chen L. Li J. Y. Yu Y. H.. (2019). Dual RNA-seq of *Xanthomonas oryzae* pv. Oryzicola infecting rice reveals novel insights into bacterial-plant interaction. PLoS One 14:e0215039. doi: 10.1371/journal.pone.0215039, PMID: 30995267 PMC6469767

[ref136] Liao S. Zhang Y. Pan X. Zhu F. Jiang C. Liu Q.. (2019). Antibacterial activity and mechanism of silver nanoparticles against multidrug-resistant *Pseudomonas aeruginosa*. Int. J. Nanomedicine 14, 1469–1487. doi: 10.2147/IJN.S191340, PMID: 30880959 PMC6396885

[ref137] Little B. J. Wagner P. A. (1997). “Succession in microfouling” in Fouling Organisms of the Indian Ocean: Biology and Control Technology. eds. Nagabhushanam R. Thompson M. (New Delhi: Oxford and IBH), 105–134.

[ref138] Liu M. Ding X. Wang X. Li J. Yang H. Yin Y. (2019). Extraction of DNA from complex biological sample matrices using guanidinium ionic liquid modified magnetic nanocomposites. RSC Adv. 9, 23119–23128. doi: 10.1039/C9RA01505A, PMID: 35514470 PMC9067247

[ref139] LoCoco P. M. Boyd J. T. Espitia Olaya C. M. Furr A. R. Garcia D. K. Weldon K. S.. (2020). Reliable approaches to extract high-integrity RNA from skin and other pertinent tissues used in pain research. PAIN Reports 5:e818. doi: 10.1097/PR9.0000000000000818, PMID: 32440611 PMC7209822

[ref140] Lu H. Que Y. Wu X. Guan T. Guo H. (2019). Metabolomics deciphered metabolic reprogramming required for biofilm formation. Sci. Rep. 9:13160. doi: 10.1038/s41598-019-49603-1, PMID: 31511592 PMC6739361

[ref141] Machata S. Müller M. M. Lehmann R. Sieber P. Panagiotou G. Carvalho A.. (2020). Proteome analysis of bronchoalveolar lavage fluids reveals host and fungal proteins highly expressed during invasive pulmonary aspergillosis in mice and humans. Virulence 11, 1337–1351. doi: 10.1080/21505594.2020.1824960, PMID: 33043780 PMC7549978

[ref142] Maghini D. G. Moss E. L. Vance S. E. Bhatt A. S. (2021). Improved high-molecular-weight DNA extraction, nanopore sequencing and metagenomic assembly from the human gut microbiome. Nat. Protoc. 16, 458–471. doi: 10.1038/s41596-020-00424-x, PMID: 33277629 PMC8750633

[ref143] Mahieu N. G. Genenbacher J. L. Patti G. J. (2016). A roadmap for the XCMS family of software solutions in metabolomics. Curr. Opin. Chem. Biol. 30, 87–93. doi: 10.1016/j.cbpa.2015.11.009, PMID: 26673825 PMC4831061

[ref144] Mahnert A. Verseux C. Schwendner P. Koskinen K. Kumpitsch C. Blohs M.. (2021). Microbiome dynamics during the HI-SEAS IV mission, and implications for future crewed missions beyond earth. Microbiome 9:27. doi: 10.1186/s40168-020-00959-x, PMID: 33487169 PMC7831191

[ref145] Mann M. Kulak N. A. Nagaraj N. Cox J. (2013). The coming age of complete, accurate, and ubiquitous proteomes. Mol. Cell 49, 583–590. doi: 10.1016/j.molcel.2013.01.029, PMID: 23438854

[ref146] Manousi N. Rosenberg E. Deliyanni E. Zachariadis G. A. Samanidou V. (2020). Magnetic solid-phase extraction of organic compounds based on Graphene oxide Nanocomposites. Molecules 25:1148. doi: 10.3390/molecules25051148, PMID: 32143401 PMC7179219

[ref147] Marotz C. Amir A. Humphrey G. Gaffney J. Gogul G. Knight R. (2018). DNA extraction for streamlined metagenomics of diverse environmental samples 62, 290–293. doi: 10.2144/000114559, PMID: 28625159

[ref148] Marvasi M. Cavalieri D. Mastromei G. Casaccia A. Perito B. (2019). Omics technologies for an in-depth investigation of biodeterioration of cultural heritage. Int. Biodeterior. Biodegradation 144:104736. doi: 10.1016/j.ibiod.2019.104736

[ref149] Meffray A. Perrin M. Richier A. Schmitt A. Ardagna Y. Biagini P. (2019). Molecular detection of Treponema pallidum subspecies pallidum in 150-year-old foetal remains, southeastern France. J. Med. Microbiol. 68, 761–769. doi: 10.1099/jmm.0.000978, PMID: 30994442

[ref150] Monds R. D. O’Toole G. A. (2009). The developmental model of microbial biofilms: ten years of a paradigm up for review. Trends Microbiol. 17, 73–87. doi: 10.1016/j.tim.2008.11.001, PMID: 19162483

[ref151] Monge M. Abdel-Hady A. Aslett L. D. Calfee M. W. Williams B. Ratliff K.. (2021). Inactivation of MS2 bacteriophage on copper film deployed in high touch areas of a public transport system. Lett. Appl. Microbiol. 74, 405–410. doi: 10.1111/lam.13624, PMID: 34862976 PMC8935140

[ref152] Monticolo F. Palomba E. Termolino P. Chiaiese P. de Alteriis E. Mazzoleni S.. (2020). The role of DNA in the extracellular environment: a focus on NETs, RETs and biofilms. Front. Plant Sci. 11:589837. doi: 10.3389/fpls.2020.589837, PMID: 33424885 PMC7793654

[ref153] Moros G. Chatziioannou A. C. Gika H. G. Raikos N. Theodoridis G. (2017). Investigation of the derivatization conditions for GC–MS metabolomics of biological samples. Bioanalysis 9, 53–65. doi: 10.4155/bio-2016-0224, PMID: 27921459

[ref154] Müller T. Kalxdorf M. Longuespée R. Kazdal D. N. Stenzinger A. Krijgsveld J. (2020). Automated sample preparation with SP3 for low-input clinical proteomics. Mol. Syst. Biol. 16:e9111. doi: 10.15252/msb.20199111, PMID: 32129943 PMC6966100

[ref155] Nagler M. Insam H. Pietramellara G. Ascher-Jenull J. (2018). Extracellular DNA in natural environments: features, relevance and applications. Appl. Microbiol. Biotechnol. 102, 6343–6356. doi: 10.1007/s00253-018-9120-4, PMID: 29858957 PMC6061472

[ref156] Nakai K. Horton P. (1999). PSORT: a program for detecting sorting signals in proteins and predicting their subcellular localization. Trends Biochem. Sci. 24, 34–35. doi: 10.1016/s0968-0004(98)01336-x, PMID: 10087920

[ref157] Nastasijevic I. Milanov D. Velebit B. Djordjevic V. Swift C. Painset A.. (2017). Tracking of listeria monocytogenes in meat establishment using whole genome sequencing as a food safety management tool: a proof of concept. Int. J. Food Microbiol. 257, 157–164. doi: 10.1016/j.ijfoodmicro.2017.06.015, PMID: 28666130

[ref158] Nesvizhskii A. I. (2014). Proteogenomics: concepts, applications and computational strategies. Nat. Methods 11, 1114–1125. doi: 10.1038/nmeth.3144, PMID: 25357241 PMC4392723

[ref159] Ni Y. Yu G. Chen H. Deng Y. Wells P. M. Steves C. J.. (2020). M2IA: a web server for microbiome and metabolome integrative analysis. Bioinformatics 36, 3493–3498. doi: 10.1093/bioinformatics/btaa188, PMID: 32176258

[ref160] Nobori T. Velásquez A. C. Wu J. Kvitko B. H. Kremer J. M. Wang Y.. (2018). Transcriptome landscape of a bacterial pathogen under plant immunity. PNAS 115, E3055–E3064. doi: 10.1073/pnas.1800529115, PMID: 29531038 PMC5879711

[ref161] Noirot-Gros M.-F. Forrester S. Malato G. Larsen P. E. Noirot P. (2019). CRISPR interference to interrogate genes that control biofilm formation in Pseudomonas fluorescens. Sci. Rep. 9:15954. doi: 10.1038/s41598-019-52400-5, PMID: 31685917 PMC6828691

[ref162] Noirot-Gros M.-F. Shinde S. Larsen P. E. Zerbs S. Korajczyk P. J. Kemner K. M. (2018). Dynamics of Aspen roots colonization by pseudomonads reveals strain-specific and mycorrhizal-specific patterns of biofilm formation. Front. Microbiol. 9:et al.:853. doi: 10.3389/FMICB.2018.00853 PMC5943511 29774013

[ref163] Ollinik J. E. Chua C. C. Brunswick P. Elner R. W. Blajkevitch O. Kim M.. (2021). Assessing diatom-mediated fatty acids in intertidal biofilm: a new conservation concern. Environ Syst Res 10:30. doi: 10.1186/s40068-021-00236-2

[ref164] Orth J. D. Thiele I. Palsson B. O. (2010). What is flux balance analysis? Nat Biotech 28, 245–248. doi: 10.1038/nbt.1614, PMID: 20212490 PMC3108565

[ref165] Paix B. Carriot N. Barry-Martinet R. Greff S. Misson B. Briand J. F.. (2020). A multi-Omics analysis suggests links between the differentiated surface Metabolome and epiphytic microbiota along the Thallus of a Mediterranean seaweed Holobiont. Front. Microbiol. 11:494. doi: 10.3389/fmicb.2020.00494, PMID: 32269559 PMC7111306

[ref166] Paix B. Othmani A. Debroas D. Culioli G. Briand J.-F. (2019). Temporal covariation of epibacterial community and surface metabolome in the Mediterranean seaweed holobiont *Taonia atomaria*. Environ. Microbiol. 21, 3346–3363. doi: 10.1111/1462-2920.14617, PMID: 30945796

[ref167] Pang Z. Chong J. Zhou G. de Lima Morais D. A. Chang L. Barrette M.. (2021). MetaboAnalyst 5.0: narrowing the gap between raw spectra and functional insights. Nucleic Acids Res. 49, W388–W396. doi: 10.1093/nar/gkab382, PMID: 34019663 PMC8265181

[ref168] Parrot D. Blümel M. Utermann C. Chianese G. Krause S. Kovalev A.. (2019). Mapping the surface microbiome and metabolome of brown seaweed *Fucus vesiculosus* by amplicon sequencing, integrated metabolomics and imaging techniques. Sci. Rep. 9:1061. doi: 10.1038/s41598-018-37914-8, PMID: 30705420 PMC6355876

[ref169] Pérez-Brocal V. Magne F. Ruiz-Ruiz S. Ponce C. A. Bustamante R. Martin V. S.. (2020). Optimized DNA extraction and purification method for characterization of bacterial and fungal communities in lung tissue samples. Sci. Rep. 10:17377. doi: 10.1038/s41598-020-74137-2, PMID: 33060634 PMC7562954

[ref170] Pieterse C. M. J. de Jonge R. Berendsen R. L. (2016). The soil-borne supremacy. Trends Plant Sci. 21, 171–173. doi: 10.1016/j.tplants.2016.01.018, PMID: 26853594

[ref171] Pluskal T. Castillo S. Villar-Briones A. Orešič M. (2010). MZmine 2: modular framework for processing, visualizing, and analyzing mass spectrometry-based molecular profile data. BMC Bioinformatics 11:395. doi: 10.1186/1471-2105-11-395, PMID: 20650010 PMC2918584

[ref172] Polman H. J. G. Jenner H. A. Bruijs M. C. M. (2020). “Technologies for biofouling control and monitoring in desalination” in Corrosion and Fouling Control in Desalination Industry. eds. Saji V. S. Meroufel A. A. Sorour A. A. (Cham: Springer)

[ref173] Polman H. Verhaart F. Bruijs M. (2013). Impact of biofouling in intake pipes on the hydraulics and efficiency of pumping capacity. Desalin. Water Treat. 51, 997–1003. doi: 10.1080/19443994.2012.707371

[ref174] Polson C. Sarkar P. Incledon B. Raguvaran V. Grant R. (2003). Optimization of protein precipitation based upon effectiveness of protein removal and ionization effect in liquid chromatography–tandem mass spectrometry. J. Chromatogr. B 785, 263–275. doi: 10.1016/S1570-0232(02)00914-5, PMID: 12554139

[ref175] Price C. W. Leslie D. C. Landers J. P. (2009). Nucleic acid extraction techniques and application to the microchip. Lab Chip 9, 2484–2494. doi: 10.1039/B907652M, PMID: 19680574

[ref176] Probst M. Ascher-Jenull J. Insam H. Gómez-Brandón M. (2021). The molecular information about deadwood Bacteriomes partly depends on the targeted environmental DNA. Front. Microbiol. 12:640386. doi: 10.3389/fmicb.2021.640386, PMID: 33986733 PMC8110828

[ref177] Pu Y. Pan J. Yao Y. Ngan W. Y. Yang Y. Li M.. (2021). Ecotoxicological effects of erythromycin on a multispecies biofilm model, revealed by metagenomic and metabolomic approaches. Environ. Pollut. 276:116737. doi: 10.1016/j.envpol.2021.116737, PMID: 33618119

[ref178] Qian C. Hettich R. L. (2017). Optimized extraction method to remove humic acid interferences from soil samples prior to microbial proteome measurements. J. Proteome Res. 16, 2537–2546. doi: 10.1021/acs.jproteome.7b00103, PMID: 28537741

[ref179] Rabinowitz J. D. Kimball E. (2007). Acidic acetonitrile for cellular Metabolome extraction from *Escherichia coli*. Anal. Chem. 79, 6167–6173. doi: 10.1021/ac070470c, PMID: 17630720

[ref180] Ram R. J. Verberkmoes N. C. Thelen M. P. Tyson G. W. Baker B. J. Blake R. C.. (2005). Community proteomics of a natural microbial biofilm. Science 308, 1915–1920. doi: 10.1126/science.1109070 15879173

[ref181] Reid A. H. Fanning T. G. Hultin J. V. Taubenberger J. K. (1999). Origin and evolution of the 1918 ‘Spanish’ influenza virus hemagglutinin gene. PNAS 96, 1651–1656. doi: 10.1073/pnas.96.4.1651, PMID: 9990079 PMC15547

[ref182] Robbe-Saule M. Babonneau J. Sismeiro O. Marsollier L. Marion E. (2017). An optimized method for extracting bacterial RNA from mouse skin tissue colonized by mycobacterium ulcerans. Front. Microbiol. 8. doi: 10.3389/fmicb.2017.00512, PMID: 28392785 PMC5364165

[ref183] Roberts L. D. Souza A. L. Gerszten R. E. Clish C. B. (2012). Targeted Metabolomics. Curr. Protoc. Mol. Biol. 98, Unit 30.2.1–Unit 30.224. doi: 10.1002/0471142727.mb3002s98, PMID: 22470063 PMC3334318

[ref184] Robinson C. Brookes S. J. Shore R. C. Kirkham J. (1998). The developing enamel matrix: nature and function. Eur. J. Oral Sci. 106, 282–291. doi: 10.1111/j.1600-0722.1998.tb02188.x, PMID: 9541238

[ref185] Rodrigues C. F. Černáková L. (2020). Farnesol and Tyrosol: secondary metabolites with a crucial quorum-sensing role in Candida biofilm development. Genes 11:444. doi: 10.3390/genes11040444, PMID: 32325685 PMC7231263

[ref186] Rohart F. Gautier B. Singh A. Lê Cao K.-A. (2017). mixOmics: an R package for ‘omics feature selection and multiple data integration. PLoS Comput. Biol. 13:e1005752. doi: 10.1371/journal.pcbi.1005752, PMID: 29099853 PMC5687754

[ref187] Röst H. L. Sachsenberg T. Aiche S. Bielow C. Weisser H. Aicheler F.. (2016). OpenMS: a flexible open-source software platform for mass spectrometry data analysis. Nat. Methods 13, 741–748. doi: 10.1038/nmeth.3959, PMID: 27575624

[ref188] Roux P.-F. Oddos T. Stamatas G. (2021). Deciphering the role of skin surface microbiome in skin health: an integrative multi-omics approach reveals three distinct metabolite-microbe clusters. J. Investig. Dermatol. 142, 469–479.e5. doi: 10.1016/j.jid.2021.07.159, PMID: 34343557

[ref189] Russotto V. Cortegiani A. Raineri S. M. Giarratano A. (2015). Bacterial contamination of inanimate surfaces and equipment in the intensive care unit. J. Intensive Care 3:54. doi: 10.1186/s40560-015-0120-5, PMID: 26693023 PMC4676153

[ref190] Rychert K. Wink L. Blohs M. Kumpitsch C. Neumann C. Moissl-Eichinger C.. (2021). Detection of microorganisms and metabolism in dune sand of a low organic content. J. Geophys. Res. Biogeo. 126:e2021JG006404. doi: 10.1029/2021JG006404

[ref191] Sadiq F. A. Yan B. Zhao J. Zhang H. Chen W. (2020). Untargeted metabolomics reveals metabolic state of *Bifidobacterium bifidum* in the biofilm and planktonic states. LWT 118:108772. doi: 10.1016/j.lwt.2019.108772

[ref192] Sauer K. (2003). The genomics and proteomics of biofilm formation. Genome Biol. 4:219. doi: 10.1186/gb-2003-4-6-219, PMID: 12801407 PMC193612

[ref193] Savojardo C. Martelli P. L. Fariselli P. Profiti G. Casadio R. (2018). BUSCA: an integrative web server to predict subcellular localization of proteins. Nucleic Acids Res. 46, W459–W466. doi: 10.1093/nar/gky320, PMID: 29718411 PMC6031068

[ref194] Sayers E. W. Cavanaugh M. Clark K. Ostell J. Pruitt K. D. Karsch-Mizrachi I. (2020). GenBank. Nucleic Acids Res. 48, D84–D86. doi: 10.1093/nar/gkz956, PMID: 31665464 PMC7145611

[ref195] Scalbert A. Brennan L. Fiehn O. Hankemeier T. Kristal B. S. van Ommen B.. (2009). Mass-spectrometry-based metabolomics: limitations and recommendations for future progress with particular focus on nutrition research. Metabolomics 5, 435–458. doi: 10.1007/s11306-009-0168-0, PMID: 20046865 PMC2794347

[ref196] Scheltema R. A. Jankevics A. Jansen R. C. Swertz M. A. Breitling R. (2011). PeakML/mzMatch: a file format, Java library, R library, and tool-chain for mass spectrometry data analysis. Anal. Chem. 83, 2786–2793. doi: 10.1021/ac2000994, PMID: 21401061

[ref197] Schiebenhoefer H. Schallert K. Renard B. Y. Trappe K. Schmid E. Benndorf D.. (2020). A complete and flexible workflow for metaproteomics data analysis based on MetaProteomeAnalyzer and Prophane. Nat. Protoc. 15, 3212–3239. doi: 10.1038/s41596-020-0368-7, PMID: 32859984

[ref198] Schrader C. Schielke A. Ellerbroek L. Johne R. (2012). PCR inhibitors – occurrence, properties and removal. J. Appl. Microbiol. 113, 1014–1026. doi: 10.1111/j.1365-2672.2012.05384.x, PMID: 22747964

[ref199] Semmouri I. De Schamphelaere K. A. C. Mees J. Janssen C. R. Asselman J. (2020). Evaluating the potential of direct RNA nanopore sequencing: Metatranscriptomics highlights possible seasonal differences in a marine pelagic crustacean zooplankton community. Mar. Environ. Res. 153:104836. doi: 10.1016/j.marenvres.2019.104836 31727392

[ref200] Seneviratne C. J. Suriyanarayanan T. Widyarman A. S. Lee L. S. Lau M. Ching J.. (2020). Multi-omics tools for studying microbial biofilms: current perspectives and future directions. Crit. Rev. Microbiol. 46, 759–778. doi: 10.1080/1040841X.2020.1828817, PMID: 33030973

[ref201] Shakya M. Lo C.-C. Chain P. S. G. (2019). Advances and challenges in Metatranscriptomic analysis. Front. Genet. 10:904. doi: 10.3389/fgene.2019.00904, PMID: 31608125 PMC6774269

[ref202] Sheng K. Cao W. Niu Y. Deng Q. Zong C. (2017). Effective detection of variation in single-cell transcriptomes using MATQ-seq. Nat. Methods 14, 267–270. doi: 10.1038/nmeth.4145, PMID: 28092691

[ref203] Sims D. Sudbery I. Ilott N. E. Heger A. Ponting C. P. (2014). Sequencing depth and coverage: key considerations in genomic analyses. Nat. Rev. Genet. 15, 121–132. doi: 10.1038/nrg3642, PMID: 24434847

[ref204] Singh A. Shannon C. P. Gautier B. Rohart F. Vacher M. Tebbutt S. J.. (2019). DIABLO: an integrative approach for identifying key molecular drivers from multi-omics assays. Bioinformatics 35, 3055–3062. doi: 10.1093/bioinformatics/bty1054, PMID: 30657866 PMC6735831

[ref205] Sinha N. van Schothorst E. M. Hooiveld G. J. E. J. Keijer J. Martins dos Santos V. A. P. Suarez-Diez M. (2021). Exploring the associations between transcript levels and fluxes in constraint-based models of metabolism. BMC Bioinformatics 22:574. doi: 10.1186/s12859-021-04488-8, PMID: 34839828 PMC8628452

[ref206] Slade E. A. Thorn R. M. S. Young A. Reynolds D. M. (2019). An in vitro collagen perfusion wound biofilm model; with applications for antimicrobial studies and microbial metabolomics. BMC Microbiol. 19:310. doi: 10.1186/s12866-019-1682-5, PMID: 31888471 PMC6937849

[ref207] Smith O. Clapham A. Rose P. Liu Y. Wang J. Allaby R. G. (2014). A complete ancient RNA genome: identification, reconstruction and evolutionary history of archaeological barley stripe mosaic virus. Sci. Rep. 4, 1–6. doi: 10.1038/srep04003, PMID: 24499968 PMC3915304

[ref208] Smith C. A. Want E. J. O’Maille G. Abagyan R. Siuzdak G. (2006). XCMS: processing mass spectrometry data for metabolite profiling using nonlinear peak alignment, matching, and identification. Anal. Chem. 78, 779–787. doi: 10.1021/ac051437y, PMID: 16448051

[ref209] Smyth G. K. (2004). Linear models and empirical Bayes methods for assessing differential expression in microarray experiments. Stat. Appl. Genet. Mol. Biol. 3, 1–25. doi: 10.2202/1544-6115.1027, PMID: 16646809

[ref210] Starke R. Jehmlich N. Bastida F. (2019). Using proteins to study how microbes contribute to soil ecosystem services: the current state and future perspectives of soil metaproteomics. J. Proteome 198, 50–58. doi: 10.1016/j.jprot.2018.11.011, PMID: 30445181

[ref211] Starr A. E. Deeke S. A. Li L. Zhang X. Daoud R. Ryan J.. (2018). Proteomic and Metaproteomic approaches to understand host–microbe interactions. Anal. Chem. 90, 86–109. doi: 10.1021/acs.analchem.7b04340, PMID: 29061041

[ref212] Stewart P. S. White B. Boegli L. Hamerly T. Williamson K. S. Franklin M. J.. (2019). Conceptual model of biofilm antibiotic tolerance that integrates phenomena of diffusion, metabolism, gene expression, and physiology. J. Bacteriol. 201:e00307-19. doi: 10.1128/JB.00307-19, PMID: 31501280 PMC6805107

[ref213] Stipetic L. H. Dalby M. J. Davies R. L. Morton F. R. Ramage G. Burgess K. E. V. (2016). A novel metabolomic approach used for the comparison of *Staphylococcus aureus* planktonic cells and biofilm samples. Metabolomics 12:75. doi: 10.1007/s11306-016-1002-0, PMID: 27013931 PMC4783440

[ref214] Stolz J. F. (2000). “Structure of microbial Mats and Biofilms” in Microbial Sediments. eds. Riding R. E. Awramik S. M. (Berlin, Heidelberg: Springer), 1–8.

[ref215] Styczynski M. P. Moxley J. F. Tong L. V. Walther J. L. Jensen K. L. Stephanopoulos G. N. (2007). Systematic identification of conserved metabolites in GC/MS data for Metabolomics and biomarker discovery. Anal. Chem. 79, 966–973. doi: 10.1021/ac0614846, PMID: 17263323

[ref216] Sugimoto M. (2021). “Capillary electrophoresis–mass spectrometry of hydrophilic metabolomics” in Metabolomics. ed. Wood P. L. (New York, NY: Springer US), 113–120.

[ref217] Sultan A. Andersen B. Svensson B. Finnie C. (2016). Exploring the plant–microbe Interface by profiling the surface-associated proteins of barley grains. J. Proteome Res. 15, 1151–1167. doi: 10.1021/acs.jproteome.5b01042, PMID: 26928395

[ref218] Sumner L. W. Lei Z. Nikolau B. J. Saito K. Roessner U. Trengove R. (2014). Proposed quantitative and alphanumeric metabolite identification metrics. Metabolomics 10, 1047–1049. doi: 10.1007/s11306-014-0739-6

[ref219] Tack I. L. M. M. Nimmegeers P. Akkermans S. Hashem I. Van Impe J. F. M. (2017). Simulation of *Escherichia coli* dynamics in biofilms and submerged colonies with an individual-based model including metabolic network information. Front. Microbiol. 8:2509. doi: 10.3389/fmicb.2017.02509, PMID: 29321772 PMC5733555

[ref220] Tang T. Xu Y. Wang J. Tan X. Zhao X. Zhou P.. (2021). Evaluation of the differences between biofilm and planktonic Brucella abortus via metabolomics and proteomics. Funct. Integr. Genomics 21, 421–433. doi: 10.1007/s10142-021-00788-7, PMID: 34009538

[ref221] Tedersoo L. Albertsen M. Anslan S. Callahan B. (2021). Perspectives and benefits of high-throughput long-read sequencing in microbial ecology. Appl. Environ. Microbiol. 87:e0062621. doi: 10.1128/AEM.00626-21, PMID: 34132589 PMC8357291

[ref222] Teng Q. Huang W. Collette T. W. Ekman D. R. Tan C. (2009). A direct cell quenching method for cell-culture based metabolomics. Metabolomics 5, 199–208. doi: 10.1007/s11306-008-0137-z

[ref223] Teufel F. Almagro Armenteros J. J. Johansen A. R. Gíslason M. H. Pihl S. I. Tsirigos K. D.. (2022). SignalP 6.0 predicts all five types of signal peptides using protein language models. Nat. Biotechnol. 40, 1023–1025. doi: 10.1038/s41587-021-01156-3, PMID: 34980915 PMC9287161

[ref224] Thaiss C. A. Levy M. Korem T. Dohnalová L. Shapiro H. Jaitin D. A.. (2016). Microbiota diurnal rhythmicity programs host Transcriptome oscillations. Cells 167, 1495–1510.e12. doi: 10.1016/j.cell.2016.11.003, PMID: 27912059

[ref225] Tian L. Wang L. (2021). Multi-omics analysis reveals structure and function of biofilm microbial communities in a pre-denitrification biofilter. Sci. Total Environ. 757:143908. doi: 10.1016/j.scitotenv.2020.143908, PMID: 33316516

[ref226] Tomlinson K. L. Lung T. W. F. Dach F. Annavajhala M. K. Gabryszewski S. J. Groves R. A.. (2021). *Staphylococcus aureus* induces an itaconate-dominated immunometabolic response that drives biofilm formation. Nat. Commun. 12:1399. doi: 10.1038/s41467-021-21718-y, PMID: 33658521 PMC7930111

[ref227] Tsugawa H. (2018). Advances in computational metabolomics and databases deepen the understanding of metabolisms. Curr. Opin. Biotechnol. 54, 10–17. doi: 10.1016/j.copbio.2018.01.008, PMID: 29413746

[ref228] Tsugawa H. Cajka T. Kind T. Ma Y. Higgins B. Ikeda K.. (2015). MS-DIAL: data-independent MS/MS deconvolution for comprehensive metabolome analysis. Nat. Methods 12, 523–526. doi: 10.1038/nmeth.3393, PMID: 25938372 PMC4449330

[ref229] Tuson H. H. Weibel D. B. (2013). Bacteria-surface interactions. Soft Matter 9, 4368–4380. doi: 10.1039/C3SM27705D, PMID: 23930134 PMC3733390

[ref230] Tyc Olaf C. Song J. S. Dickschat M. V. Garbeva P. (2017). The ecological role of volatile and soluble secondary metabolites produced by soil bacteria. Trends Microbiol. 25, 280–292. doi: 10.1016/j.tim.2016.12.002, PMID: 28038926

[ref231] Utturkar S. M. Klingeman D. M. Hurt R. A. J. Brown S. D. (2017). A case study into microbial genome assembly gap sequences and finishing strategies. Front. Microbiol. 8:1272. doi: 10.3389/fmicb.2017.01272, PMID: 28769883 PMC5513972

[ref232] van Gulik W. M. (2010). Fast sampling for quantitative microbial metabolomics. Curr. Opin. Biotechnol. 21, 27–34. doi: 10.1016/j.copbio.2010.01.008, PMID: 20149631

[ref233] Vicente F. A. Plazl I. Ventura S. P. M. Žnidaršič-Plazl P. (2020). Separation and purification of biomacromolecules based on microfluidics. Green Chem. 22, 4391–4410. doi: 10.1039/C9GC04362D

[ref234] Vickery K. Deva A. Jacombs A. Allan J. Valente P. Gosbell I. B. (2012). Presence of biofilm containing viable multiresistant organisms despite terminal cleaning on clinical surfaces in an intensive care unit. J. Hosp. Infect. 80, 52–55. doi: 10.1016/j.jhin.2011.07.007, PMID: 21899921

[ref235] Vilanova C. Porcar M. (2020). Art-omics: multi-omics meet archaeology and art conservation. Microb. Biotechnol. 13, 435–441. doi: 10.1111/1751-7915.13480, PMID: 31452355 PMC7017809

[ref236] Vitális E. Nagy F. Tóth Z. Forgács L. Bozó A. Kardos G.. (2020). Candida biofilm production is associated with higher mortality in patients with candidaemia. Mycoses 63, 352–360. doi: 10.1111/myc.13049 31943428

[ref237] Wangsanuwat C. Heom K. A. Liu E. O’Malley M. A. Dey S. S. (2020). Efficient and cost-effective bacterial mRNA sequencing from low input samples through ribosomal RNA depletion. BMC Genomics 21:717. doi: 10.1186/s12864-020-07134-4, PMID: 33066726 PMC7565789

[ref238] Weidt S. Haggarty J. Kean R. Cojocariu C. I. Silcock P. J. Rajendran R.. (2016). A novel targeted/untargeted GC-Orbitrap metabolomics methodology applied to *Candida albicans* and *Staphylococcus aureus* biofilms. Metabolomics 12:189. doi: 10.1007/s11306-016-1134-2, PMID: 28003796 PMC5097782

[ref239] Wen B. Mei Z. Zeng C. Liu S. (2017). metaX: a flexible and comprehensive software for processing metabolomics data. BMC Bioinformatics 18:183. doi: 10.1186/s12859-017-1579-y, PMID: 28327092 PMC5361702

[ref240] Weyrich L. S. Farrer A. G. Eisenhofer R. Arriola L. A. Young J. Selway C. A.. (2019). Laboratory contamination over time during low-biomass sample analysis. Mol. Ecol. Resour. 19, 982–996. doi: 10.1111/1755-0998.13011, PMID: 30887686 PMC6850301

[ref241] Whitchurch C. B. Tolker-Nielsen T. Ragas P. C. Mattick J. S. (2002). Extracellular DNA required for bacterial biofilm formation. Science 295:1487. doi: 10.1126/science.295.5559.1487 11859186

[ref242] Williamson K. S. Richards L. A. Perez-Osorio A. C. Pitts B. McInnerney K. Stewart P. S.. (2012). Heterogeneity in *Pseudomonas aeruginosa* biofilms includes expression of ribosome hibernation factors in the antibiotic-tolerant subpopulation and hypoxia-induced stress response in the metabolically active population. J. Bacteriol. 194, 2062–2073. doi: 10.1128/JB.00022-12, PMID: 22343293 PMC3318454

[ref243] Wishart D. S. (2019). NMR metabolomics: a look ahead. J. Magn. Reson. 306, 155–161. doi: 10.1016/j.jmr.2019.07.013, PMID: 31377153

[ref244] Wishart D. S. Jewison T. Guo A. C. Wilson M. Knox C. Liu Y.. (2013). HMDB 3.0—the human Metabolome database in 2013. Nucleic Acids Res. 41, D801–D807. doi: 10.1093/nar/gks1065, PMID: 23161693 PMC3531200

[ref245] Wu X. Siehnel R. J. Garudathri J. Staudinger B. J. Hisert K. B. Ozer E. A.. (2019). In vivo proteome of *Pseudomonas aeruginosa* in airways of cystic fibrosis patients. J. Proteome Res. 18, 2601–2612. doi: 10.1021/acs.jproteome.9b00122, PMID: 31060355 PMC6750005

[ref246] Wu J. Xi C. (2009). Evaluation of different methods for extracting extracellular DNA from the biofilm matrix. Appl. Environ. Microbiol. 75, 5390–5395. doi: 10.1128/AEM.00400-09, PMID: 19561191 PMC2725481

[ref247] Yang C. Chowdhury D. Zhang Z. Cheung W. K. Lu A. Bian Z.. (2021). A review of computational tools for generating metagenome-assembled genomes from metagenomic sequencing data. Comput. Struct. Biotechnol. J. 19, 6301–6314. doi: 10.1016/j.csbj.2021.11.028, PMID: 34900140 PMC8640167

[ref248] Yao L. Sheflin A. M. Broeckling C. D. Prenni J. E. (2019). “Data processing for GC-MS- and LC-MS-based untargeted Metabolomics” in High-Throughput Metabolomics: Methods and Protocols. ed. D’Alessandro A. (New York, NY: Springer), 287–299. 10.1007/978-1-4939-9236-2_18 31119670

[ref249] Yu Z. Miller H. C. Puzon G. J. Clowers B. H. (2018). Application of untargeted metabolomics for the detection of pathogenic *Naegleria fowleri* in an operational drinking water distribution system. Water Res. 145, 678–686. doi: 10.1016/j.watres.2018.09.003, PMID: 30212806

[ref250] Yu T. Park Y. Johnson J. M. Jones D. P. (2009). apLCMS—adaptive processing of high-resolution LC/MS data. Bioinformatics 25, 1930–1936. doi: 10.1093/bioinformatics/btp291, PMID: 19414529 PMC2712336

[ref251] Zeki Ö. C. Eylem C. C. Reçber T. Kır S. Nemutlu E. (2020). Integration of GC–MS and LC–MS for untargeted metabolomics profiling. J. Pharm. Biomed. Anal. 190:113509. doi: 10.1016/j.jpba.2020.113509, PMID: 32814263

[ref252] Zhang Z. Cao Y. Li Y. Chen X. Ding C. Liu Y. (2021). Risk factors and biofilm formation analyses of hospital-acquired infection of *Candida pelliculosa* in a neonatal intensive care unit. BMC Infect. Dis. 21:620. doi: 10.1186/s12879-021-06295-1, PMID: 34187390 PMC8244135

[ref253] Zhang X. Li L. Mayne J. Ning Z. Stintzi A. Figeys D. (2018). Assessing the impact of protein extraction methods for human gut metaproteomics. J. Proteome 180, 120–127. doi: 10.1016/j.jprot.2017.07.001, PMID: 28705725

[ref254] Zhang Y. Ng C. K. Cohen Y. Cao B. (2014). Cell growth and protein expression of *Shewanella oneidensis* in biofilms and hydrogel-entrapped cultures. Mol. BioSyst. 10, 1035–1042. doi: 10.1039/c3mb70520j, PMID: 24626808

[ref255] Zhang B. Powers R. (2012). Analysis of bacterial biofilms using NMR-based metabolomics. Future Med. Chem. 4, 1273–1306. doi: 10.4155/fmc.12.59, PMID: 22800371 PMC3564560

[ref256] Zhang B. Xie M. Bruschweiler-Li L. Brüschweiler R. (2018). Nanoparticle-assisted Metabolomics. Meta 8:E21. doi: 10.3390/metabo8010021, PMID: 29533993 PMC5876010

[ref257] Zhao S. Li L. (2020). Chemical derivatization in LC-MS-based metabolomics study. TrAC Trends Anal. Chem. 131:115988. doi: 10.1016/j.trac.2020.115988

[ref258] Zhao J. Wang G. Chu J. Zhuang Y. (2019). Harnessing microbial metabolomics for industrial applications. World J. Microbiol. Biotechnol. 36:1. doi: 10.1007/s11274-019-2775-x, PMID: 31811524

[ref259] Zhao L. Zhao X. Wu J. Lou X. Yang H. (2019). Comparison of metabolic response between the planktonic and air-dried *Escherichia coli* to electrolysed water combined with ultrasound by 1H NMR spectroscopy. Food Res. Int. 125:108607. doi: 10.1016/j.foodres.2019.108607, PMID: 31554111

[ref260] Zhou G. Ewald J. Xia J. (2021). OmicsAnalyst: a comprehensive web-based platform for visual analytics of multi-omics data. Nucleic Acids Res. 49, W476–W482. doi: 10.1093/nar/gkab394, PMID: 34019646 PMC8262745

[ref261] Zoetendal E. G. Ben-Amor K. Akkermans A. D. L. Abee T. de Vos W. M. (2001). DNA isolation protocols affect the detection limit of PCRApproaches of bacteria in samples from the HumanGastrointestinal tract. Syst. Appl. Microbiol. 24, 405–410. doi: 10.1078/0723-2020-00060, PMID: 11822677

